# Supplementary material for: Factors perceived to facilitate or hinder handwashing among primary students: a qualitative assessment of the Mikono Safi intervention schools in NW Tanzania
Source: BMJ Open. 2019 Nov 28;9(11):e030947. doi: 10.1136/bmjopen-2019-030947 (PMC6924754; doi:10.1136/bmjopen-2019-030947)
Supplement: Supplementary data [file bmjopen-2019-030947supp001.pdf]

STUDY GUIDES

Appendix 1: FGD/Friendship Pair Interview question guide for students

Name of District....................................... Name of School..
Age of the respondent........................... Class of the respondent.........................

A: Knowledge about HWWS and helminth infections

1. Tell me about the last time you washed your hands.
    a. What did you use?
    b. Where were you? (*School, home, friend's house, etc*.)
    c. What were you doing immediately before you washed your hands?
    d. What did you do after you washed your hands?
    e. Potential probes:
        i. Are there other times during the day you usually wash your hands?
        ii. Why is it important to wash hands then?
2. The last time you washed your hands; can you show me how you did it? (consider bringing soap and water to interview)
    a. Is that how you always wash your hands? [*Probe on any different times or ways that they describe].*
3. What happens if you don't wash your hands?[*Probe on responses]*
4. Imagine you are at school and you go to wash your hands.
    a. You find there is no soap at the handwashing station. What would you do? (*Probe on responses: Then what would you do? Would you try something else? Etc*.)
    b. Now imagine there is no water at the handwashing station. What would you do then? (Probe…)
5. Now, imagine you are at home and want to wash your hands. What do you do if there is no soap? No water?

B: Implementation of handwashing lessons

6. Please tell me about any lessons you've had in class recently about handwashing
7. What do you remember about the lesson? (Probe on as many specifics as possible.)
8. During the lesson,
    a. What did the teacher do?
    b. What did the children do?
9. What parts did you like most about the lesson? Why? What was your favourite part of the lessons?

C: Availability and accessibility of Handwashing materials

10. While in school, where do you go to wash your hands?
11. Are there times when you want to wash your hands but water is finished? If yes, tell me about the last time you went but the water was finished. (Probe on how long ago, what time of day, what they did in response.) How often does this happen?
12. Are there times when you need soap to wash your hands but soap is finished? Same as above.

D: Effectiveness of Mikono Safi in targeting in motivational and emotional drivers of handwashing with soap

13. During the handwashing exercise that we talked about earlier, teachers used some examples,
    a. What are the examples?
    b. Which of the examples do you remember most?
    c. Why do you remember that example?
14. [Bring and use some of the motivational messages during the interviews; Show the children the Muta and Koku flip chart then ask]
    a. Please tell me what you see on the flip chart
    b. What do you think about Muta?
    c. How does Muta's poor hygiene behavior make you feel?
    d. What do think about Koku?
    e. How does Koku's good hygiene make you feel?
    f. Imagine Muta was you young brother, what would to help him?
15. Show the children show the children the side by the side poster
    a) What do you see on the poster? [Probe on the response]
    b) Focusing on the side of the child with worms
        i. What is the girl/boy doing wrong?
        ii. How does the girl/boy's behavior make you feel?
        iii. What would you do if you were this girl/boy?
        iv. What would you help if this boy/girl was your little brother/sister?
16. If you saw child from standard one coming from the toilet and running to play, what would you do? Why?
17.

E: Consistence in message delivery

18. Which day of the week do you usually receive the hand washing lessons?
19. What time of the day do the teachers come for hand washing lessons?
20. What activities do children participate in during handwashing lessons?

Okello E, *et al. BMJ Open* 2019; 9:e030947. doi: 10.1136/bmjopen-2019-030947

Appendix 2: In-depth Interview guide for Teachers/school staff

Name of District.. Name of School...
Age of the respondent............................ Education level of the respondent..........................

A: Knowledge about HWWS and helminth infections

1. In your opinion, how much do children know about when and how to washing hands? [*probe on response*]
2. What do you think motivates them to wash hands?
3. What do you think discourages from washing hands at critical times?
4. In your observation, If for example, children want to wash hands but there is no water, what do they usually do?
5. In your observation, If for example, children want to wash hands but there is no water, what do they usually do?

B: Lessons implemented and appropriateness of the lessons

6. Please tell me how handwashing lessons are delivered in your school
   a. What has been your role in delivering these lessons?
   b. In what classes are the lessons delivered?
   c. How often are these lessons delivered?
   d. What activities are the children involved in during these lessons?
   e. What has been your experience in delivering these lessons?
7. How would you describe the involvement of teachers and school staff in delivering handwashing lessons in your school? [*probe on the answer*]
8. What motivates teachers to get involved in delivering handwashing lessons?
9. What discourages teachers from getting involved in delivering handwashing lessons
10. What is your opinion about the contents of the handwashing curriculum?
11. How far if the curriculum is able address knowledge and skill gaps related to handwashing in children?
12. Do you have any recommendations to improve the handwashing curriculum?

C: Availability and accessibility of Handwashing materials

13. Tell me about the availability of water and soap in your school
   a. How does the school ensure water is available for children to wash hands?
   b. How does the school ensure soap is available for children to wash hands?
14. Are there times when water is not available for children to wash hands? How often does this happen?
15. Are there times when soap is finished? How often does this happen?
16. How does the school maintain the handwashing stand?

17. How does your school ensure that latrines are kept clean? [*probe for who is involved*]
18. How does your school ensure there are sufficient and appropriate supplies for latrine cleaning?
19. How are latrines managed and maintained?[probe about who is involved]

D: Effectiveness of Mikono Safi in targeting in motivational and emotional drivers of handwashing with soap

20. How would you describe children response to handwashing lessons?
21. Among the motivational messages used in the handwashing curriculum, what parts do children enjoy most during handwashing lessons? Why?
22. In your view, motivational messages; what parts do children remember most about handwashing lessons? Why?
23. What changes have you observed in children's handwashing behavior?
    a. Is there a difference between girls and boys?
    b. Are there differences among age groups?

Okello E, *et al. BMJ Open* 2019; 9:e030947. doi: 10.1136/bmjopen-2019-030947
