# Supplementary material for: Factors perceived to facilitate or hinder handwashing among primary students: a qualitative assessment of the Mikono Safi intervention schools in NW Tanzania
Source: BMJ Open. 2019 Nov 28;9(11):e030947. doi: 10.1136/bmjopen-2019-030947 (PMC6924754; doi:10.1136/bmjopen-2019-030947)
Supplement: Author's manuscript [file bmjopen-2019-030947.draft_revisions.pdf]

BMJ Open is committed to open peer review. As part of this commitment we make the peer review history of every article we publish publicly available.

When an article is published we post the peer reviewers' comments and the authors' responses online. We also post the versions of the paper that were used during peer review. These are the versions that the peer review comments apply to.

The versions of the paper that follow are the versions that were submitted during the peer review process. They are not the versions of record or the final published versions. They should not be cited or distributed as the published version of this manuscript.

BMJ Open is an open access journal and the full, final, typeset and author-corrected version of record of the manuscript is available on our site with no access controls, subscription charges or pay-per-view fees (http://bmjopen.bmj.com).

If you have any questions on BMJ Open's open peer review process please email info.bmjopen@bmj.com

BMJ Open

Factors perceived to facilitate or hinder handwashing among primary students: a qualitative assessment of the Mikono Safi intervention schools in NW Tanzania

Journal:	*BMJ Open*
Manuscript ID	bmjopen-2019-030947
Article Type:	Research
Date Submitted by the Author:	09-Apr-2019
Complete List of Authors:	Okello, Elialilia; Mwanza Intervention Trials Unit, MITU;<br>Kapiga, Saidi ; London School of Hygiene and Tropical Medicine, Infectious Disease Epidemiology<br>Grosskurth, Heiner; London School of Hygiene and Tropical Medicine, Epidemiology and Public Health<br>Makata, Kenneth; Mwanza Intervention Trials Unit, MITU<br>Mcharo, Onike; Mwanza Intervention Trials Unit, MITU<br>Kinungh'i, Safari; National Insitute for Medcial Research, Mwanza Centre<br>Dreibelbis , Robert; London School of Hygiene and Tropical Medicine
Keywords:	QUALITATIVE RESEARCH, COM B MODEL, HANDWASHING

SCHOLARONE™
Manuscripts

Factors perceived to facilitate or hinder handwashing among primary students: a qualitative assessment of the Mikono Safi intervention schools in NW Tanzania

Authors:

1. Elialilia Okello*, Mwanza intervention Trials Unit, P. O Box 11936, Mwanza Tanzania; E-mail addresses: Elialilia.okello@mitu.or.tz; elly16.sp@gmail.com
2. Saidi Kapiga: Mwanza Intervention Trials unit, Mwanza Tanzania & London School of Hygiene and Tropical Medicine, London UK
3. Heiner Grosskurth: Mwanza Intervention Trials unit, Mwanza Tanzania & London School of Hygiene and Tropical Medicine, London UK
4. Kenneth Makata: Mwanza Intervention Trials unit, Mwanza Tanzania
5. Onike Mcharo: Mwanza Intervention Trials unit, Mwanza Tanzania
6. Safari Kinungh'i: Mwanza Intervention Trials unit, & National Institute for Medical Research, Mwanza Centre, Tanzania
7. Robert Dreibelbis: London School of Hygiene and Tropical Medicine, London UK

Word count: 5,273

Corresponding Author

For peer review only - http://bmjopen.bmj.com/site/about/guidelines.xhtml

Abstract

Objective: To explore the success of a school-based WASH intervention in supporting positive behaviour change among students using an integrated perspective that links implementation fidelity, school-level compliance, and individual-level drivers of targeted emotional behavior.

Design: The study was a longitudinal qualitative assessment of barriers and facilitators to handwashing using in-depth interview with teachers, focus group discussions and Friendship pair interview with students.

Setting: The study was conducted in 4 purposively selected intervention schools in three districts of Bukoba urban, Bukoba rural and Muleba in Kagera region, North Western Tanzania.

Participants: Participants were 16 teachers aged between 23 and 52 years and 100 students aged between 7 and 15 years.

Results: Overall, the Mikono Safi interventions had a positive effect on student reported capability and motivation for HWWS. Improvements in handwashing skills and knowledge attributed to the Mikono Safi intervention were reported by both teachers and students. The improvement was a result of participatory learning approach to hand hygiene education. It was also noted that the intervention was successful in targeting the emotional drivers of behaviour such as disgust, fear, and nurture through various messaging platforms

For peer review only - http://bmjopen.bmj.com/site/about/guidelines.xhtml

and activities. Similarly, the installation of robust handwashing facilities improved physical opportunity for handwashing. The engagement of students and teachers in the intervention activities had also creates a school community that valued and supported handwashing. However, factors outside schools' control such as dry season often disrupted water supply.

Conclusion: The intervention was successful in improving motivation and capability for handwashing, but improving handwashing opportunity-consistent availability remained a challenge.

***Key words**: Handwashing, COM B Model, qualitative research*

For peer review only

Strengths and limitations of this study

- In-depth interviews, FGDs and FPI were well suited methods to examine barriers and facilitators to appropriate handwashing behaviour, and were essential for the success of the intervention from the perspectives of students and teachers
- Interviews were conducted with graduate social scientists with prior experience in qualitative methods
- Sustained contact with students and teachers during the two rounds of data collection helped to reduce the risk of social desirability bias
- This was a qualitative study involving a small purposively selected sample of schools, teachers and pupils and findings are primarily based on individual interviews and FGDs.

For peer review only

Introduction

Soil-transmitted helminth (STH) infections are among the most prevalent infections worldwide [1], with demonstrable negative impacts on child development [2-4]. Current control strategies focus on school-based mass drug administration (MDA) programs [5]. Although, MDA remain a cost-effective way to reduce the burden of STHs, recent research has shown that school-based MDA programs alone can still result in rapid re-infection when water, sanitation and hygiene (WASH) conditions remain poor [6, 7]. A recent randomized controlled trial in China demonstrated that the promotion of handwashing with soap in schools can result in significant reductions in STH prevalence [8], suggesting that school-based WASH interventions could play a significant role in STH infection control strategies. This is the rationale underlying the current Mikono Safi study (Protocol #: ISRCTN45013173), a cluster randomized trial of a school-based handwashing intervention in western Tanzania. The trial will assess the impact of a school-based handwashing and hygiene promotion intervention on STH reinfection among primary school-aged children.

School-based WASH interventions in low- and middle-income countries are associated with a range of health and educational outcomes, including: diarrhea, respiratory infection, school attendance and health outcomes in the domestic environment [9-14]. However, multiple studies have shown that the impact of school-based WASH interventions are often variable[15, 16]. For the most part, this variability has been viewed through the lens of *intervention fidelity* and *compliance* – the extent to which schools and/or implementing organizations have provided WASH services in schools routinely and in a manner consistent with intervention protocols [15, 17]. This perspective is largely based within the disciplines of implementation science. However, focusing on the implementation of school-based

For peer review only - http://bmjopen.bmj.com/site/about/guidelines.xhtml

interventions has the potential to ignore the more complex relationships between implementation fidelity and changes in the more proximal determinants of individual pupil WASH behaviors. While studies have documented or tested the extent to which school WASH interventions have resulted in changes in specific hygiene behaviours [10, 18], the mechanisms through which interventions change behaviours remains underexplored. Further, few studies examine intervention feasibility and acceptability from a child's perspective.

The goal of our study was to explore the success of a school-based WASH intervention in supporting positive behaviour change among students using an integrated perspective that links implementation fidelity, school-level compliance, and individual-level psychosocial drivers of targeted behavior. We provide an overview of the design, justification, and implementation of the Mikono Safi intervention and explore the success of the Mikono Safi intervention in changing routine handwashing with soap (HWWS) among children attending intervention schools.

Our analysis is informed by the COM-B framework [19] which posits that behaviour is determined by three main factors: capability, opportunity, and motivation. Capability refers to an individual's psychological and physical capacity to complete HWWS. This includes skills, knowledge related to the behavior, self-efficacy and proper social and educational support. Opportunity refers to factors external to the individual that make HWWS possible or prompt it. For the purposes of the Mikono Safi intervention, this includes access to soap and water and access to clean latrines. Motivation is defined as the mental processes that direct HWWS. This can include cognitive, rational thought processes, habits, and emotional responses that prompt HWWS.

For peer review only - http://bmjopen.bmj.com/site/about/guidelines.xhtml

Methods

Study setting and Intervention Development

In 2009, the Tanzanian government began annual school-based MDA for STM control. Kagera, a region situated in the north-western part of Tanzania, west of Lake Victoria has been one of the major focus for government MDA initiatives [20]. School children of this area have received annual treatment with albendazole for the past eight years. But recent survey data on the distribution of STH in Tanzania shows that the prevalence of these infections has remained high [21].

The Mikono Safi intervention was designed to increase handwashing with soap among school-aged children in an attempt to sustain the effects of routine deworming campaigns. In this cluster-randomized trial, a school-based water, sanitation, and hygiene (WASH) intervention has been implemented following albendazole administration in 8 randomly selected schools and STH reinfection rates compared to 8 schools in a randomly selected control arm. Intervention development was informed by the Behaviour Centred Design process [22] and combined a number of evidence-based and theoretically-informed elements to foster and trigger handwashing behaviours at schools. Intervention components were field tested and iteratively refined over a period of three months prior to implementation.

The Mikono Safi intervention has multiple, integrated component. Formative research identified key motives [22] associated with HWWS for pupils, including: disgust, nurture, and fear. These motives were reflected in two key characters Koku, a young girl attending schools, and Muta, a young boy pupil. The characters of Koku and Muta were central to all

For peer review only - http://bmjopen.bmj.com/site/about/guidelines.xhtml

messaging activities. Characters and motives were linked with HWWS through a number of posters in the school, stories that were told during health education sessions, and game packages delivered to children. Message were delivered through a range of classroom and play-based activities that also included an educational component designed to teach children about disease risk and hygiene in a fun and interactive manner [23, 24]. In each of the intervention school, students received health education classroom sessions delivered at the beginning of the intervention with two follow-up “booster” sessions scheduled for 6 and 12 months respectively. These education sessions focused on handwashing; its importance, when to wash hands, worm infection and materials used for handwashing. The sessions were delivered to complement the existing science curriculum which covered general body hygiene and sanitation with very limited emphasis to handwashing.

Some of the intervention schools, especially the rural based schools did not have any form handing washing facilities while urban based schools had basic tippy-taps. To improve access to water for HWWS, intervention schools were provided with new robust handwashing stations consisted of 100-litre water containers placed close to the toilet in areas accessible to children with visible paths connecting the toilets to handwashing stations [**Figure 1**]. We also added a number of “nudges” to new facilities, including iconography and marked paths connecting latrines to handwashing facilities [18, 25].

[*Insert Figure 1 here*]

Organizational support was an important part of the Mikono Safi intervention. In addition to the larger infrastructure improvements, each school was provided with 100 bars of soap

For peer review only - http://bmjopen.bmj.com/site/about/guidelines.xhtml

every month. At each school, discussions were held with teachers to ensure soap was reliably made available for students to wash hands, and teachers were trained on how to implement handwashing lessons. During the handwashing education sessions, teachers guided students to practice a correct way of washing ones. All intervention development was done in collaboration with District Education Office, who supported the intervention.

School-level interventions were supplemented with key activities targeting parents to foster community support and to influence WASH behaviours in the home environment. In brief, results from pre-intervention stool sample analyses were shared with parents in an organized manner and parents were given information on simple actions that households could take to support behaviours that protect against STH. A manuscript with further details on the parental intervention and its effect on behaviours in the home is forthcoming.

Study Design, Sample and Data Collection Methods

This was a longitudinal qualitative study conducted in 4 intervention schools. These 4 schools were purposively selected to include 2 urban and 2 rural schools. In each school, we conducted two rounds of data collection. The first round of data was collected in late April 2018; two months after the intervention had been implemented in the 4 schools. The second round was collected in the last week of October 2018, six months after round 1 of data collection and 8 months after the intervention had been rolled out in the four schools. The qualitative data were completed before the quantitative end-line survey for the trial started.

In the first round of data collection, we completed 2 focus group discussions (FDGs) in each school, for a total of 8 focus group discussions (FGDs) with a total of 60 students. The FGDs

For peer review only - http://bmjopen.bmj.com/site/about/guidelines.xhtml

were age and gender segregated – resulting in 2 FGDs for older boys, 2 FGDs for older girls, 2 FGDs for younger boys, and 2 FGDs for younger girls. To assess students' handwashing skills, at the end of each FGD, the moderator walked with the participating students to the handwashing stations for students to demonstrate how they wash hands. Following FGDs, we conducted 4 interviews with teachers at participating schools. Interviews covered: information on teachers' perception about students' knowledge regarding the relationship between HWW and STH infection; experience with implementing handwashing lessons, availability and accessibility of handwashing materials at school, and perceived effectiveness of the intervention in targeting motivational and emotional drivers for handwashing.

For the second round of data collection (October 2018), we completed 5 friendship pair interviews (FPIs) at each school, for a total of 10 respondents per school. FPI is a method used to collect data from children where the target respondent is encouraged to come to the interview with a friend with the intention of creating a conducive, non-threatening environment for the children [28]. The research team, with support from respective class teachers, solicited student volunteers. In each class, one student was selected. The selected student was asked to request his/her best friend to join him or her to talk to the research team about handwashing. FPIs collected information on students' knowledge regarding the relationship between HWW and STH infection; perception about implementation of handwashing lessons including consistence in delivery of handwashing messages, availability and accessibility of handwashing materials at school, and the effectiveness of the intervention in targeting motivational and emotional drivers for handwashing. We also completed semi-structured interviews with 4 teachers per school. Selected teachers include

For peer review only

For peer review only - http://bmjopen.bmj.com/site/about/guidelines.xhtml

the school head teacher, 1 teacher in charge of sanitation and hygiene and 2 other teachers who are involved in implementing hand hygiene education.

Due to prior planning and clear explanation of the study purpose there were no refusals. All the teachers and students that were requested to participate in the study agreed and took part in the study. Data was collected using pilot tested FGD and FPI guides. All the interviews and focus group discussions were conducted in private empty rooms that provided by the school administration. Each FGD/FPI lasted between 1 hour 30 minutes and 2 hours while interview with teachers lasted between 30 and 45 minutes.

Research team

Data collection team included the first, fourth and fifth authors and two research assistants. The two research assistants included 1 male and 1 female with bachelor's degree in social science and experience in qualitative research methods. Prior to starting fieldwork the research assistants received additional one week's training in qualitative research methods. The first author, with PhD in social science and an experienced qualitative researcher participated in actual data collection at the beginning each data collection round and coordinate the study. At the beginning of each round of data collection the study team visited schools, explained the purpose of the study, discussed study activities with the school administration and made appointments for interviews and focus group discussions

Public involvement

This qualitative study is assessing an intervention that was preceded by a rigorous formative research. During the formative research there was extensive involvement of students,

For peer review only - http://bmjopen.bmj.com/site/about/guidelines.xhtml

teachers and other stakeholders in testing the various components of the intervention. The feedback received from these stakeholders informed the implementation of the intervention. Similarly, the interview and FGD guides used in the qualitative study were pilot-tests and revised to include feedback from the participants. The findings from the qualitative study will be disseminated through stakeholders meetings.

Data analysis

Audio recorded interviews and FGDs were transcribed in Kiswahili and translated into English and combined with field notes ready for analysis. Data were analyzed using Atlas.ti version 7, a qualitative data analysis and research software

The COM-B conceptual framework served as a guide for data analysis [29] and was used to define topics and subtopics explored in data collection and themes explored in analysis. For analysis, the first author read all interview and FGD transcripts and formulated draft codes based on recurring accounts and descriptions that were identified in the transcripts. The first and last authors reviewed, discussed and refined the codes and agreed on the final list of codes that were used for analysis. Related codes were combined into themes and relevant data extracts were collated and within themes identified [30, 31]. Thematic analysis approach was used to facilitate the structuring, description and interpretation of themes [32]. Key findings are described below according to the COM-B components: Capability (knowledge and skills); opportunity (availability and accessibility of handwashing facilities, water and soap, and network of people who valued and encouraged handwashing) and motivation (ability to make decision and take action for handwashing, and emotional responses that trigger handwashing).

For peer review only - http://bmjopen.bmj.com/site/about/guidelines.xhtml

For peer review only

For peer review only - http://bmjopen.bmj.com/site/about/guidelines.xhtml

Results

A total of 20 FPIs and 8 FGDs were conducted with students. Ages of participants in both FPIs and FGDs ranged from 7 to 15 years. Sixteen teachers participated in two rounds of interviews and each teacher was interviewed twice. Teachers who participated in the interviews were aged between 23 and 52 years.

Capability

We defined capability as the having necessary skills and competencies to complete the behaviour. Overall, the Mikono Safi interventions had a positive effect on student reported capability for HWWS. Capability consisted of two major sub-themes that emerged from data collection, improvements in handwashing skills and knowledge attributed to the Mikono Safi intervention

Handwashing skills: Students indicated in both FGDs and FPIs that because they had been involved and had taken an active role in the learning about HWWS. Participation in the handwashing demonstrations they were able to acquire the necessary handwashing skills. During interviews and FGDs students volunteered to demonstrate handwashing and they were able to follow the correct steps involved.

> *The teacher took us to the handwashing stations and demonstrated to us on how to wash hands. We were told that when you come from the toilet you wet your hands, apply soap then scrub* **(FGD older boys, school 2)**

Both teachers and students believed that the acquired skills were important in facilitating handwashing.

For peer review only - http://bmjopen.bmj.com/site/about/guidelines.xhtml

Handwashing lessons and educational messaging: Both children and teachers indicated that the intensive handwashing program and use of educational messaging had greatly improved children's knowledge about how and when to wash hands. All the children in both the FGDs and FPIs recalled receiving at least two lessons specific to handwashing in additional to general sanitation and hygiene education provided within the school curriculum under the science lesson. Students understood the link between hand hygiene and infections. They also knew the key times they were required to wash hands.

> *There are some changes in students' handwashing behavior. Now they always wash their hands especially after using the toilet. The changes have been as a result of the ongoing project activities, through which the children have been taught about the importance of handwashing. Now they know so they have now built a habit of washing hands, which was not the case before this project came to us* **(IDI teacher 2 school 1**)

> *Before project came we had some tippy taps near the toilets. These were small and you had to step on them for water to pour on your hands. We did not always wash hands after using the toilet. But now were are motivated because we have been educated on the importance of handwashing so we now wash our hands always* **(FPI younger girls school)**

Opportunity

For peer review only - http://bmjopen.bmj.com/site/about/guidelines.xhtml

From our data, the Mikono Safi intervention influenced handwashing through changing the physical opportunity for handwashing and altering the social environment related to handwashing at schools.

Physical Opportunity: Teachers and students alike indicated that availability and location of the handwashing facilities were important facilitators of handwashing. Teachers in particular noted that every child had to pass handwashing stations on their way to and from the latrine. Handwashing stations were visible and difficult to ignore:

> *Given the location of the handwashing stations… on the way out of the toilet, everybody who uses the toilet can see them on their way out…but you know children like to play with water, so making water and soap available for children makes handwashing part of a game (IDI,* **Teacher 2, school 3** *)*

> *Availability and positioning of handwashing facilities and a clear path leading to the toilets facilitated handwashing and encouraged students to wash hand especially after using the toilet.* **(IDI, Teacher 2, school 1)**

> *…We wash our hands at the handwashing stations near the toilet. Before they installed the handwashing tanks we never use to wash hands because we did not have water to wash hands but these days we wash our hands because they have put the tanks. But in the past we never used to wash hands (***FPI, younger girls, school 3***)*

Children in the FGDs indicated that the improved handwashing facilities were not only easier and more convenient to use when compared to tip-taps that the school was using before but also the tanks contained much more water allowing larger number of students to have access to water for washing hands.

For peer review only - http://bmjopen.bmj.com/site/about/guidelines.xhtml

"…Before the installation of the handwashing tanks we were using tip-taps to wash hands and we had only few of them, the water would get finished quickly. But they were even difficult to use. With the current handwashing tanks you go there and open the tap and wash hands. The tanks are big and many students are able to wash hands using water in the tanks. ***(FGD, older boys, school 1****)*

Beyond the physical availability of handwashing facilities, the most important facilitator to handwashing was the consistent provision of supplies (water and soap). In some schools, especially schools with a large student population, it was not uncommon for water to get used up early in the day - particularly during break time when the majority of the children used the toilet. Schools were able to mitigate these interruptions in water availability in various ways including creating roster for fetching water. Although creation of rosters for fetching water was not part of the intervention, schools that had well supervised and well implemented class rosters for fetching water well had more children indicating that water was routinely available at the handwashing station.

*…there are times when water gets finished, but in our school we have a teacher and students responsible for health and hygiene. So when water is finished from the tanks we inform the teacher or student leader and they send a group of students to find water (***FPI, older girls, school 2)**

Water availability, however, was often determined by factors outside of the school's direct control. All schools experienced disruptions in water supply to varying degrees. In three of the four schools in the study, this was a problem that occurred during the morning break when majority of the students used the toilets. In one school it was a problem that occurred frequently – multiple times during a week. During these periods of limited

For peer review only - http://bmjopen.bmj.com/site/about/guidelines.xhtml

availability, teachers needed to interrupt classes for certain groups of students in order for them to fill the tanks. Often, teachers were unwilling to interrupt classes, especially in schools that did not have a well-established class roster for refilling water tanks. Lack of water had a more negative influence on handwashing behaviours particularly if water was unavailable for more than one day in a week.

In general, both students and teachers described that soap was not consistently provided for washing hands. This was primarily an issue of internal distribution rather than supply/availability issues. In one of the schools, for example, there was no soap at the handwashing station when the moderator took the students to the handwashing station for handwashing demonstration. Students noted that they had reported to the responsible teacher earlier but no soap had been provided. However, when the moderator sent a student to the head teacher's office to request for soap, the soap was provided. Similarly, discussions with school administrations indicated that soap was usually available in the school storage even when provision was inconsistent:

> *"There are a number of times during the week when soap is not available at the handwashing station. This can happen like three times in a week. If the soap is not at the handwashing station we usually go to the Head teacher's office to get the soap"* (**FGD, younger girls, school 3**)

> *There are times when the soap is not available because the teacher responsible did not check or the students have not reported. This happens sometimes but that can usually is for few hours in a day not the whole day* (IDI, **Teacher 1, school 2)**

For peer review only - http://bmjopen.bmj.com/site/about/guidelines.xhtml

Today in the morning, we found no soap at the handwashing station so we washed our hands without soap. But we reported to the prefect responsible for ensuring the soap is available, he went and reported to the teacher. I went to the toilet on two days ago and there was also no soap in the afternoon **(FGD, older girls, school 2)**

Social opportunity: Data suggest that the Mikono Safi intervention had created school communities that valued handwashing and that teachers were willing to support handwashing activities through teaching and reminding children to wash hands, supervising children to fill the water tanks, and ensuring the soap was available. Students on the other hand were willing to participate in activities that ensured water and soap were available at the handwashing stations:

*"If I came out of the toilet and there is no water I will pick a bucket get water from the main reserve tank and fill the handwashing tanks so that other children and I can wash our hands" (***FGD, Older girls, school 2***)*

.

*"Today I went to the toilet in the morning but there was no soap at the handwashing station at that time. I reported to the student in charge of sanitation and s/he promised to inform the teacher that there was not soap at the handwashing station" (***FPI, older girls, school 1***)*

Students especially the youngest ones were keen to report colleagues that did not wash hands after using the toilet. Teachers described experiences where students would report other students who did not wash hands to teachers.

For peer review only - http://bmjopen.bmj.com/site/about/guidelines.xhtml

*"Certainly, there are changes in children's handwashing behavior. I am a teacher for the pre-primary class (the youngest group in school). What I have observed is that these days these young ones report colleagues that do not wash hands after using the toilet. If one of them used a toilet and did not wash hands the others will come running to you to report him or her; they will come and tell you 'teacher, so and so did not wash his/her hands after using the toilet'; they give you such reports (***IDI, teacher, school 2***)*

Motivation

Emotional triggers: The Mikono Safi intervention was designed to target the emotions of disgust, fear, and nurture through various messaging platforms and activities. Data suggest that the intervention was successful in targeting these emotional drivers of behaviour. Most children found the suggestion that if you do not wash hands you may be ingesting faecal matter extremely repulsive and disgusting. In particular, multiple children described Muta – the boy character depicted in the educational materials as a boy who did not know how to wash his hands - as repulsive and disgusting. On the other hand children found Koku (portrayed as a hygiene loving child) attractive and every child interviewed indicated that he or she would like be like Koku, clean and smart, but also kind and willing to teach a fellow student who may not have knowledge of handwashing.

I feel bad about Muta's behavior; the fact that he did not wash his hand makes me feel disgusted…. but we like Koku because she was clean and she knew how to wash

For peer review only - http://bmjopen.bmj.com/site/about/guidelines.xhtml

hands in order to protect herself from disease. Everyone would like to be like Koku, she is clean, smart and she likes helping others….the teacher told us anybody who does not wash hands after using the toilet is Muta (**FGD, older girls, school 4**)

*…what I remember most is what the teacher told us during the handwashing lessons… the teachers told us that if you use the toilet but you do not wash your hands and you go and eat anything you may be eating feces. So when you think about that you remember to wash your hands and you will remind all your friends to wash hands (**FGD, older girls, school 1**)*

Level of involvement of students in decision: Although use of class rosters for fetching water was not part of the intervention, multiple schools used rosters to ensure water was available at the handwashing facilities. For some schools this activity was students lead and for others it was teacher lead activity. For example, in schools that had active students' health clubs, members of these clubs were charged with the responsible for supervising all health related activities in schools. Hence, schools which had active students' health clubs developed the roster for health activities of each day of the week.

Students in schools that had student leaders involved in decision making about availability of water and soap were more motivated to take action to ensure they have materials to wash hand. Students in schools with active student participation were more likely to do something if water was unavailable at the handwashing stations. When children believed that they had power to influence their own handwashing practices – or when students had agency - they were willing to take an active role in activities that would facilitate handwashing among themselves and others.

For peer review only - http://bmjopen.bmj.com/site/about/guidelines.xhtml

*"The students have received the handwashing lessons very well. They wash hand but they also encourage others to wash hands. For example if they saw a colleague who is dirty they encourage him/her to wash hand in order to avoid being labeled Muta, character depicted in some of the education materials of a dirty boy was using a shield to protect himself from germ" (***IDI, teacher 1, school 3***)*

Changes over time: There was no difference between data collection round one and two in terms of capability opportunity and motivation for handwashing. In round two of data collection students demonstrated good knowledge and skills for handwashing. Similarly, the opportunity for handwashing remained largely unchanged between the data collection round one and two. The installed handwashing facilities were well maintained in all the four schools. The schools continued to experienced disruptions in water supply and success in mitigating the disruption varied from school to school.

Discussion

This component of the study was set out to describe the barriers and facilitators to handwashing behaviour from the perspectives of teachers and students and the success of the Mikono Safi intervention on addressing the determinants of behaviour. Our results show that improved handwashing skills and knowledge, social and physical opportunities for handwashing and appropriate emotional drivers are important facilitators to handwashing.

All the students interviewed indicated knowing when, how and why one should wash hands. Although insufficient on its own, knowledge may be a necessary precursor for HWWS [33]. It is important to note that education and health messaging included in the Mikono Safi were

For peer review only - http://bmjopen.bmj.com/site/about/guidelines.xhtml

not abstract – it was focused around the risk of preventing intestinal worms. Studies in India have shown that providing individuals with specific- and actionable health information can improve WASH behaviours [34]. Having lessons that focus on hand hygiene and its risk with specific and known health outcomes in addition to regular sanitation and hygiene education may be a promising option for facilitating handwashing behaviour among school children.

Our health messaging also had an impact on the emotional drivers of hygiene behaviours. Posters and information went beyond just health messaging and included elements of disgust and fear. Data suggest that we were successful in triggering emotional responses in children through our information campaigns. Studies have shown how motivational drivers can be successful in changing hygiene behaviours [35], although only a limited number of studies have explored handwashing motives as it relates to children ([36] While previous studies have utilized motivation-based messaging in isolation our study shows how these can be further combined with targeted health education to influence both motivation and capability. This may be a critical component of hygiene promotion efforts, particularly when new or novel health outcomes are targeted or potentially negative emotions such as disgust and fear are the primary drivers targeted by the intervention.

Nurture also emerged as an important driver of behaviour, one that we were successful in targeting with messaging. Students spoke fondly of the fact Koku (the female student in education materials) helped her friends out; and both teachers and students discussed the ways that the school had provided a supportive environment for handwashing. This is not the first study to identify nurture as an important determinant of children's behaviour. A study in Bangladesh that used covert video cameras to record handwashing behaviour

For peer review only - http://bmjopen.bmj.com/site/about/guidelines.xhtml

found numerous instances of older children helping younger children washing hands and modelling positive behaviours for one another [37]. Students in our study responded positively to the nurture messages and suggest that positive peer pressures are a potential avenue for improving HWWS in schools.

Making handwashing facilities visible, difficult to ignore but also convenient were among the attributes mentioned as important by both teachers and students. However, beyond the physical positioning and convenience, the most important facilitator to handwashing was consistent provision of water and soap. Although all schools experienced disruptions in water supply especially at peak hours, some schools that were able to implementing a well supervised class rosters for fetching water succeeded to avoid interruptions in water availability at handwashing stations. Similarly students and teachers suggested that inconsistency in the availability of soap at the handwashing stations was largely an issue of internal distribution rather than supply issues. Multiple studies have documented institutional barriers to the consistent provision of hygiene materials in schools [26, 38]. Our data suggests that while the Mikono Safi intervention was successful in changing capability and motivation, opportunity may not have been adequately improved by the intervention in some of the schools. Having school-level systems in place – such as a detailed schedule for classrooms to collect water – did allow schools to mitigate some of the larger environmental factors that limit water availability in schools. Schools with detailed duty rosters in place were also more successful in maintaining consistent provision of handwashing materials.

For peer review only - http://bmjopen.bmj.com/site/about/guidelines.xhtml

There are a few limitations that need to be considered when interpreting the results of this study. This was a qualitative study involving a small sample of schools, teachers and pupils. Findings are primarily based on individual interviews and FGDs. Data from direct observation of students' behaviour was not collected during the qualitative study. However, in-depth interviews, FGDs and FPI were well suited methods to examine barriers and facilitators to appropriate handwashing behavior, and were essential for the success of the intervention from the perspectives of students and teachers.

In conclusion, the COM-B Framework provided a prudent way to explore the success of the Mikono Safi intervention in addressing the barriers and facilitators for handwashing in our study population. In general, we found that the intervention was successful in improving motivation for handwashing and capability for handwashing, but improving handwashing opportunity remains a challenge.

For peer review only

For peer review only - http://bmjopen.bmj.com/site/about/guidelines.xhtml

References

1. Brooker, S., A.C.A. Clements, and D.A.P. Bundy, *Global epidemiology, ecology and control of soil-transmitted helminth infections.* Advances in parasitology, 2006. **62**: p. 221-261.

2. Drake, L., et al., *Geohelminth infections (Ascariasis, Trichuriasis and Hookworm): cognitive and developmental impacts.* Seminars in Pediatric Infectious Diseases, 2000. **11**: p. 245-251

3. Hotez, P.J., et al., *Hookworm Infection.* New England Journal of Medicine, 2004. **351**(8): p. 799-807.

4. Stephenson, L., M. Latham, and E. Ottesen, *Malnutrition and parasitic helminth infections. . 2000;121(Suppl):S23–S38.* Parasitology, 2000. **121**(Supplement): p. S23-S38.

5. Fenwick, A., et al., *Drugs for the control of parasitic diseases: current status and development in schistosomiasis.* Trends in Parasitology, 2003. **19**: p. 509-515.

6. WHO, *Soil-transmitted helminth infections*, in *Fact sheet* 2013b, World Health Organization.

7. Yap, P., et al., *Rapid Re-Infection with Soil-Transmitted Helminths after Triple-Dose Albendazole Treatment of School-Aged Children in Yunnan, People's Republic of China.* Am J Trop Med Hyg, 2013. **89**(1): p. 23-31.

8. Bieri , F.A., et al., *Health-Education Package to Prevent Worm Infections in Chinese Schoolchildren.* New England Journal of Medicine, 2013. **368**(17): p. 1603-1612.

9. Chard, A.N., et al., *The impact of school water, sanitation, and hygiene improvements on infectious disease using serum antibody detection.* PLoS neglected tropical diseases, 2018. **12**(4): p. e0006418.

10. Caruso, B.A., et al., *Assessing the impact of a school-based latrine cleaning and handwashing program on pupil absence in Nyanza Province, Kenya: a cluster-randomized trial.* Tropical medicine & international health, 2014. **19**(10): p. 1185-1197.

11. Dreibelbis, R., et al., *The impact of school water, sanitation, and hygiene interventions on the health of younger siblings of pupils: a cluster-randomized trial in Kenya.* American journal of public health, 2014. **104**(1): p. e91-e97.

12. Dreibelbis, R., et al., *Water, sanitation, and primary school attendance: a multi-level assessment of determinants of household-reported absence in kenya.* International Journal of Educational Development, 2013. **33**(5): p. 457-465.

13. Freeman, M.C., et al., *The impact of a school-based water supply and treatment, hygiene, and sanitation programme on pupil diarrhoea: a cluster-randomized trial.* Epidemiol Infect, 2014. **142**(2): p. 340-51.

14. Freeman, M.C., et al., *Assessing the impact of a school-based water treatment, hygiene and sanitation programme on pupil absence in Nyanza Province, Kenya: a cluster-randomized trial.* Trop Med Int Health, 2012. **17**(3): p. 380-91.

For peer review only - http://bmjopen.bmj.com/site/about/guidelines.xhtml

15. Garn, J.V., et al., *The Role of Adherence on the Impact of a School-Based Water, Sanitation, and Hygiene Intervention in Mali.* The American journal of tropical medicine and hygiene, 2017. **96**(4): p. 984-993.

16. Trinies, V., et al., *The Impact of a School-Based Water, Sanitation, and Hygiene Program on Absenteeism, Diarrhea, and Respiratory Infection: A Matched–Control Trial in Mali.* The American journal of tropical medicine and hygiene, 2016. **94**(6): p. 1418-1425.

17. Chard, A.N. and M.C. Freeman, *Design, Intervention Fidelity, and Behavioral Outcomes of a School-Based Water, Sanitation, and Hygiene Cluster-Randomized Trial in Laos.* International journal of environmental research and public health, 2018. **15**(4): p. 570.

18. Dreibelbis, R., et al., *Behavior Change without Behavior Change Communication: Nudging Handwashing among Primary School Students in Bangladesh.* International journal of environmental research and public health, 2016. **13**(1): p. 129.

19. Michie, S., M.M. van Stralen, and R. West, *The behaviour change wheel: A new method for characterising and designing behaviour change interventions.* Implementation Science : IS, 2011. **6**: p. 42-42.

20. Tanzania;, M., *National Master Plan for Neglected Tropical Diseases.* , T. Ministry of Health and Social Welfare, Editor. 2009.

21. GAHI, *Distribution of soil-transmitted helminth survey data in Tanzania*. 2017, Global Atlas of helminth infections.

22. Aunger, R. and V. Curtis, *Behaviour centred design: towards an applied science of behaviour change.* Health psychology review, 2016. **10**(4): p. 425-446.

23. Golinkoff, D.G.S.R.M. and K. Hirsh-Pasek, *Play= Learning: How play motivates and enhances children's cognitive and social-emotional growth*. 2006: Oxford University Press.

24. Kapp, K.M., *The gamification of learning and instruction: game-based methods and strategies for training and education*. 2012: John Wiley & Sons.

25. Grover, E., et al., *Comparing the behavioural impact of a nudge-based handwashing intervention to high-intensity hygiene education: a cluster-randomised trial in rural Bangladesh.* Tropical medicine & international health, 2018. **23**(1): p. 10-25.

26. Alexander, K., et al., *Improving service delivery of water, sanitation, and hygiene in primary schools: a cluster-randomized trial in western Kenya.* Journal of Water and Health, 2013. **11**(2): p. 507-519.

27. Organization, W.H., *Bench aids for the diagnosis of intestinal parasites.* 1994.

28. Wilson, A.D., A.J. Onwuegbuzie, and L.P. Manning, *Using paired depth interviews to collect qualitative data.* The Qualitative Report, 2016. **21**(9): p. 1549-1573.

For peer review only

For peer review only - http://bmjopen.bmj.com/site/about/guidelines.xhtml

29. Michie, S., M.M. Van Stralen, and R. West, *The behaviour change wheel: a new method for characterising and designing behaviour change interventions.* Implementation science, 2011. **6**(1): p. 42.

30. Braun, V. and V. Clarke, *Using thematic analysis in psychology.* Qualitative research in psychology, 2006. **3**(2): p. 77-101.

31. Ritchie, J., L. Spencer, and W. O'Connor, *Carrying out qualitative analysis.* Qualitative research practice: A guide for social science students and researchers, 2003. **2003**: p. 219-62.

32. Attride-Stirling, J., *Thematic networks: an analytic tool for qualitative research.* Qualitative research, 2001. **1**(3): p. 385-405.

33. Coombes, Y. and J. Devine, *Introducing FOAM: A Framework to Analyze Handwashing Behaviors to Design Effective Handwashing Programs; World Bank*, W.a.S. Program, Editor 2010, World Bank: Washington, DC.

34 Trent, M., et al., *Access to Household Water Quality Information Leads to Safer Water: A Cluster Randomized Controlled Trial in india.* Environmental science & technology, 2018. **52**(9): p. 5319-5329.

35. Biran, A., et al., *Effect of a behaviour-change intervention on handwashing with soap in India (SuperAmma): a cluster-randomised trial.* The Lancet Global Health, 2014. **2**(3): p. e145-e154.

36. Watson, J, Dreibelbis, R Aunger, R; Deola, C ; King, K; Long, S; Rachel P. Chased, RP; Cumming, O. Ch*ild's play: Harnessing play and curiosity motives to improve child handwashing in a humanitarian setting*. International Journal of Hygiene and Environmental Health, https://doi.org/10.1016/j.ijheh.2018.09.002

37. Witte, K., *Putting the fear back into fear appeals: The extended parallel process model.* Communications Monographs, 1992. **59**(4): p. 329-349.

38. Grover, E., et al., *Social influence on handwashing with soap: results from a cluster randomized controlled trial in Bangladesh.* The American journal of tropical medicine and hygiene, 2018. **99**(4): p. 934-936.

For peer review only

For peer review only - http://bmjopen.bmj.com/site/about/guidelines.xhtml

Footnotes

Acknowledgments: We thank John Robert and Winnie Muriba for assisting with data collection and transcription of the interviews and focus group discussions. We are grateful to the parents for allowing their children to take part in the study and the school administration of the 4 schools for giving us permission to talk to students and teachers. We are grateful to the study participants-- students and teachers for their time and contributions to the study

Contributors: EO conceived the study and was the investigator with overall responsibility for the management of the study. EO and RD were responsible for the original design and protocol. SK, HG, SK, contributed to the study design methodology and obtaining funding. KM and OM supervised fieldwork and reviewed the draft manuscript. EO analyzed the data and wrote the first draft of the manuscript. RD, HG, SK, and SK provided a critical review for important intellectual content. All authors were involved in the final version of the manuscript and approved the manuscript for submission.

Disclaimer: The views expressed are those of the authors and not necessarily those of the

Legend:
Figure 1: Robust handwashing facility with "nudges" installed in the intervention schools

For peer review only

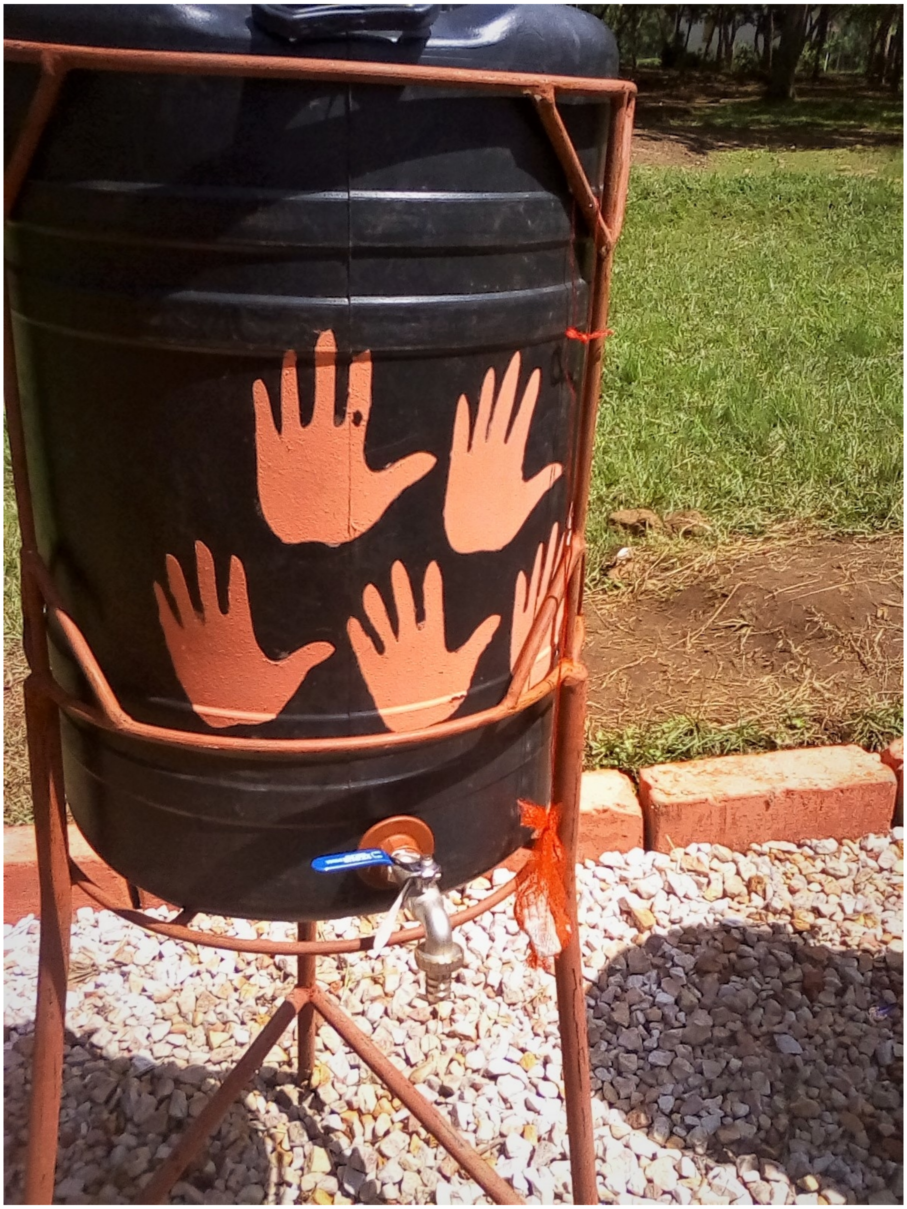

Robust handwashing facility with “nudges” installed in the intervention schools

423x564mm (72 x 72 DPI)

STUDY GUIDES

Appendix 1: Friendship Pair Interview guide for students

Name of District....................................... Name of School.......................................
Age of the respondent........................... Class of the respondent.........................

A: Knowledge about HWWS and helminth infections

1. Tell me about the last time you washed your hands.
    a. What did you use?
    b. Where were you? (*School, home, friend's house, etc*.)
    c. What were you doing immediately before you washed your hands?
    d. What did you do after you washed your hands?
    e. Potential probes:
        i. Are there other times during the day you usually wash your hands?
        ii. Why is it important to wash hands then?
2. The last time you washed your hands; can you show me how you did it? (consider bringing soap and water to interview)
    a. Is that how you always wash your hands? [*Probe on any different times or ways that they describe].*
3. What happens if you don't wash your hands?[*Probe on responses]*
4. Imagine you are at school and you go to wash your hands.
    a. You find there is no soap at the handwashing station. What would you do? (*Probe on responses: Then what would you do? Would you try something else? Etc*.)
    b. Now imagine there is no water at the handwashing station. What would you do then? (Probe…)
5. Now, imagine you are at home and want to wash your hands. What do you do if there is no soap? No water?

B: Implementation of handwashing lessons

6. Please tell me about any lessons you've had in class recently about handwashing
7. What do you remember about the lesson? (Probe on as many specifics as possible.)
8. During the lesson,
    a. What did the teacher do?
    b. What did the children do?
9. What parts did you like most about the lesson? Why? What was your favourite part of the lessons?

For peer review only - http://bmjopen.bmj.com/site/about/guidelines.xhtml

C: Availability and accessibility of Handwashing materials

10. While in school, where do you go to wash your hands?
11. Are there times when you want to wash your hands but water is finished? If yes, tell me about the last time you went but the water was finished. (Probe on how long ago, what time of day, what they did in response.) How often does this happen?
12. Are there times when you need soap to wash your hands but soap is finished? Same as above.

D: Effectiveness of Mikono Safi in targeting in motivational and emotional drivers of handwashing with soap

13. During the handwashing exercise that we talked about earlier, teachers used some examples,
    a. What are the examples?
    b. Which of the examples do you remember most?
    c. Why do you remember that example?
14. [Bring and use some of the motivational messages during the interviews; Show the children the Muta and Koku flip chart then ask]
    a. Please tell me what you see on the flip chart
    b. What do you think about Muta?
    c. How does Muta's poor hygiene behavior make you feel?
    d. What do think about Koku?
    e. How does Koku's good hygiene make you feel?
    f. Imagine Muta was you young brother, what would to help him?
15. Show the children show the children the side by the side poster
    a) What do you see on the poster? [Probe on the response]
    b) Focusing on the side of the child with worms
        i. What is the girl/boy doing wrong?
        ii. How does the girl/boy's behavior make you feel?
        iii. What would you do if you were this girl/boy?
        iv. What would you help if this boy/girl was your little brother/sister?
16. If you saw child from standard one coming from the toilet and running to play, what would you do? Why?
17.

E: Consistence in message delivery

18. Which day of the week do you usually receive the hand washing lessons?
19. What time of the day do the teachers come for hand washing lessons?
20. What activities do children participate in during handwashing lessons?

For peer review only - http://bmjopen.bmj.com/site/about/guidelines.xhtml

Appendix 2: In-depth Interview guide for Teachers/school staff

Name of District....................................... Name of School.......................................
Age of the respondent............................ Education level of the respondent.........................

A: Knowledge about HWWS and helminth infections

1. In your opinion, how much do children know about when and how to washing hands? [*probe on response*]
2. What do you think motivates them to wash hands?
3. What do you think discourages from washing hands at critical times?
4. In your observation, If for example, children want to wash hands but there is no water, what do they usually do?
5. In your observation, If for example, children want to wash hands but there is no water, what do they usually do?

B: Lessons implemented and appropriateness of the lessons

6. Please tell me how handwashing lessons are delivered in your school
   a. What has been your role in delivering these lessons?
   b. In what classes are the lessons delivered?
   c. How often are these lessons delivered?
   d. What activities are the children involved in during these lessons?
   e. What has been your experience in delivering these lessons?
7. How would you describe the involvement of teachers and school staff in delivering handwashing lessons in your school? [*probe on the answer*]
8. What motivates teachers to get involved in delivering handwashing lessons?
9. What discourages teachers from getting involved in delivering handwashing lessons
10. What is your opinion about the contents of the handwashing curriculum?
11. How far if the curriculum is able address knowledge and skill gaps related to handwashing in children?
12. Do you have any recommendations to improve the handwashing curriculum?

C: Availability and accessibility of Handwashing materials

13. Tell me about the availability of water and soap in your school
    a. How does the school ensure water is available for children to wash hands?
    b. How does the school ensure soap is available for children to wash hands?
14. Are there times when water is not available for children to wash hands? How often does this happen?
15. Are there times when soap is finished? How often does this happen?
16. How does the school maintain the handwashing stand?

For peer review only - http://bmjopen.bmj.com/site/about/guidelines.xhtml

17. How does your school ensure that latrines are kept clean? [*probe for who is involved*]
18. How does your school ensure there are sufficient and appropriate supplies for latrine cleaning?
19. How are latrines managed and maintained?[probe about who is involved]

D: Effectiveness of Mikono Safi in targeting in motivational and emotional drivers of handwashing with soap

20. How would you describe children response to handwashing lessons?
21. Among the motivational messages used in the handwashing curriculum, what parts do children enjoy most during handwashing lessons? Why?
22. In your view, motivational messages; what parts do children remember most about handwashing lessons? Why?
23. What changes have you observed in children's handwashing behavior?
    a. Is there a difference between girls and boys?
    b. Are there differences among age groups?

For peer review only

COREQ (COnsolidated criteria for REporting Qualitative research) Checklist

A checklist of items that should be included in reports of qualitative research. You must report the page number in your manuscript where you consider each of the items listed in this checklist. If you have not included this information, either revise your manuscript accordingly before submitting or note N/A.

Topic	Item No.	Guide Questions/Description	Reported on Page No.
**Domain 1: Research team and reflexivity**			
Personal characteristics			
Interviewer/facilitator	1	Which author/s conducted the interview or focus group?	9
Credentials	2	What were the researcher's credentials? E.g. PhD, MD	9
Occupation	3	What was their occupation at the time of the study?	9
Gender	4	Was the researcher male or female?	9
Experience and training	5	What experience or training did the researcher have?	9
Relationship with participants			
Relationship established	6	Was a relationship established prior to study commencement?	9
Participant knowledge of the interviewer	7	What did the participants know about the researcher? e.g. personal goals, reasons for doing the research	9
Interviewer characteristics	8	What characteristics were reported about the inter viewer/facilitator? e.g. Bias, assumptions, reasons and interests in the research topic	9
**Domain 2: Study design**			
Theoretical framework			
Methodological orientation and Theory	9	What methodological orientation was stated to underpin the study? e.g. grounded theory, discourse analysis, ethnography, phenomenology, content analysis	4, 9, 10
Participant selection			
Sampling	10	How were participants selected? e.g. purposive, convenience, consecutive, snowball	7
Method of approach	11	How were participants approached? e.g. face-to-face, telephone, mail, email	9
Sample size	12	How many participants were in the study?	7 & 10
Non-participation	13	How many people refused to participate or dropped out? Reasons?	9
Setting			
Setting of data collection	14	Where was the data collected? e.g. home, clinic, workplace	9
Presence of non-participants	15	Was anyone else present besides the participants and researchers?	9
Description of sample	16	What are the important characteristics of the sample? e.g. demographic data, date	10
Data collection			
Interview guide	17	Were questions, prompts, guides provided by the authors? Was it pilot tested?	9
Repeat interviews	18	Were repeat inter views carried out? If yes, how many?	8
Audio/visual recording	19	Did the research use audio or visual recording to collect the data?	9
Field notes	20	Were field notes made during and/or after the inter view or focus group?	9
Duration	21	What was the duration of the inter views or focus group?	9
Data saturation	22	Was data saturation discussed?	—
Transcripts returned	23	Were transcripts returned to participants for comment and/or	—

For peer review only

Topic	Item No.	Guide Questions/Description	Reported on Page No.
		correction?	
**Domain 3: analysis and findings**			
Data analysis			
Number of data coders	24	How many data coders coded the data?	10
Description of the coding tree	25	Did authors provide a description of the coding tree?	—
Derivation of themes	26	Were themes identified in advance or derived from the data?	10
Software	27	What software, if applicable, was used to manage the data?	9
Participant checking	28	Did participants provide feedback on the findings?	—
Reporting			
Quotations presented	29	Were participant quotations presented to illustrate the themes/findings? Was each quotation identified? e.g. participant number	11-18
Data and findings consistent	30	Was there consistency between the data presented and the findings?	11-18
Clarity of major themes	31	Were major themes clearly presented in the findings?	11-18
Clarity of minor themes	32	Is there a description of diverse cases or discussion of minor themes?	11-18

Developed from: Tong A, Sainsbury P, Craig J. Consolidated criteria for reporting qualitative research (COREQ): a 32-item checklist for interviews and focus groups. *International Journal for Quality in Health Care*. 2007. Volume 19, Number 6: pp. 349 – 357

Once you have completed this checklist, please save a copy and upload it as part of your submission. DO NOT include this checklist as part of the main manuscript document. It must be uploaded as a separate file.

For peer review only

BMJ Open

Factors perceived to facilitate or hinder handwashing among primary students: a qualitative assessment of the Mikono Safi intervention schools in NW Tanzania

Journal:	*BMJ Open*
Manuscript ID	bmjopen-2019-030947.R1
Article Type:	Original research
Date Submitted by the Author:	21-Aug-2019
Complete List of Authors:	Okello, Elialilia; Mwanza Intervention Trials Unit, MITU; National Institute for Medical Research (NIMR), Mwanza Centre<br>Kapiga, Saidi ; London School of Hygiene and Tropical Medicine, Infectious Disease Epidemiology; Mwanza Intervention Trials Unit<br>Grosskurth, Heiner; London School of Hygiene and Tropical Medicine, Epidemiology and Public Health; NIMR, Mwanza Centre<br>Makata, Kenneth; Mwanza Intervention Trials Unit, MITU; NIMR, Mwanza Centre<br>Mcharo, Onike; Mwanza Intervention Trials Unit, MITU; NIMR, Mwanza Centre<br>Kinungh'i, Safari; National Insitute for Medcial Research, Mwanza Centre<br>Dreibelbis , Robert; London School of Hygiene and Tropical Medicine
**Primary Subject Heading**:	Public health
Secondary Subject Heading:	Qualitative research
Keywords:	Handwashing, COM-B Model, QUALITATIVE RESEARCH

SCHOLARONE™
Manuscripts

Factors perceived to facilitate or hinder handwashing among primary students: a qualitative assessment of the Mikono Safi intervention schools in NW Tanzania

Authors:

1. Elialilia Okello*, Mwanza Intervention Trials Unit (MITU), P. O Box 11936, Mwanza & National Institute for Medical Research (NIMR), Mwanza Centre, Tanzania; E-mail addresses: elialilia.okello@mitu.or.tz; elly16.sp@gmail.com

2. Saidi Kapiga: Mwanza Intervention Trials Unit, Mwanza Tanzania & London School of Hygiene and Tropical Medicine (LSHTM), London UK & NIMR, Mwanza Centre, Tanzania

3. Heiner Grosskurth: Mwanza Intervention Trials Unit, Mwanza & London School of Hygiene and Tropical Medicine, London UK & NIMR, Mwanza Centre, Tanzania

4. Kenneth Makata: Mwanza Intervention Trials Unit, Mwanza & NIMR, Mwanza Centre, Tanzania

5. Onike Mcharo: Mwanza Intervention Trials Unit, Mwanza & NIMR, Mwanza Centre, Tanzania

6. Safari Kinungh'i: National Institute for Medical Research, Mwanza Centre, Tanzania

7. Robert Dreibelbis: London School of Hygiene and Tropical Medicine, London UK

****Corresponding Author***

For peer review only - http://bmjopen.bmj.com/site/about/guidelines.xhtml

Abstract

Objective: To qualitatively assess the effects of a multi-modal school-based water, sanitation, and hygiene (WASH) intervention on handwashing behaviour among primary students in NW Tanzania.

Design: The study was a qualitative assessment of barriers and facilitators to handwashing among students attending primary schools participating in the Mikono Safi Trial (Kiswahili for 'Clean Hands; trial registration: ISRCTN45013173), a cluster-randomized trial assessing the impact of a school-based WASH intervention on selected soil transmitted helminth infections. Data collection methods included in-depth interviews with teachers, focus group discussions and friendship pair interviews with students. The Capability-Opportunity-Motivation and Behaviour (COM-B) model was used to inform data collection and analysis.

Setting: The study was conducted in 4 purposively selected intervention schools in three districts of Kagera region, North Western Tanzania (Bukoba urban, Bukoba rural and Muleba dsitricts).

Participants: Participants comprised 16 purposively selected teachers aged between 23 and 52 years and 100 students aged 7 to 15 years

Results: The Mikono Safi intervention increased students' reported capability and motivation to wash their hands with soap at key times, particularly after visiting the toilet. Improvements in students' handwashing knowledge and skills were reported by both

For peer review only - http://bmjopen.bmj.com/site/about/guidelines.xhtml

teachers and students, and motivation for hand washing was enhanced by emotional drivers such as disgust, fear and nurture. Newly established handwashing stations improved the physical opportunity to wash hands, although the availability of water and the provision of soap was not always consistent (e.g. due to internal organisational shortcomings or during the dry season). Students and teachers were actively engaged in intervention implementation which created a school community that valued and supported improved hand hygiene.

Conclusion: The intervention was successful in improving capability and motivation for handwashing. Handwashing opportunity was also greatly improved, although the supply with water and soap was sometimes interrupted, calling for much stronger multi-sectoral collaboration to improve access to water at schools.

For peer review only

***Key words**: Handwashing, COM B Model, qualitative research*

For peer review only - http://bmjopen.bmj.com/site/about/guidelines.xhtml

Strengths and limitations of this study

- In-depth interviews of teachers and focus group discussions and indepth friendship pair interviews proved to be suitable methods to examine barriers to and facilitators of appropriate handwashing behaviour.
- These methods generated consistent results suggesting that the Mikono Safi intervention was effective in improving handwashing behaviour
- This was a qualitative study involving a small purposively selected sample of schools, teachers and pupils and may therefore not be representative for all 8 intervention schools participating in the trial or for other primary schools in Tanzania.

For peer review only

Introduction

Soil-transmitted helminth (STH) infections are among the most prevalent infections worldwide, affecting over 1.5 billion people [1] . These infections have demonstrable negative impacts on child development [2, 3] . Current control strategies focus on school-based mass drug administration (MDA) programmes. Since 2006 the World Health Organisation recommends annual deworming among all at-risk people living in endemic areas if the population prevalence of soil-transmitted helminth infections exceeds 20%, and twice a year if it exceeds 50%, using orally perscribed albendazole (400 mg) or mebendazole (500 mg) which are both deemed effective, inexpensive and easy to administer by non-medical personnel (e.g. teachers) [4] . In 2009, the Tanzanian government began school-based MDA for STH control. Kagera, a region situated in north-western Tanzania west of Lake Victoria, has been a major focus for this MDA initiative [5] . School children of this area have received annual treatment with albendazole for the past eight years. However, recent survey data on the distribution of STH in Tanzania shows that the prevalence of these infections has remained high [6] , which is in keeping with recent research showing rapid re-infection following MDA programmes when water, sanitation and hygiene (WASH) conditions remain poor [7] . A recent randomized controlled trial in China demonstrated that the promotion of handwashing with soap in schools can result in significant reductions in STH prevalence [8], suggesting that school-based WASH interventions could play a significant role in STH infection control.

The Mikono Safi (Swahili for “clean hands”) intervention was designed to increase handwashing with soap (HWWS) among school-aged children in an attempt to sustain the effects of routine MDA. It is a multi-modal intervention, combining health education, games

For peer review only - http://bmjopen.bmj.com/site/about/guidelines.xhtml

and stories targeting key motivational drivers, improvements in school handwashing infrastructure, and training and support to school staff. Training for teachers in all the intervention school was done by a qualified public health specialist and a social scientist with a Master's degree in sociology. Further details are provided below. The impact of the Mikono Safi intervention is being assessed in an ongoing cluster-randomized trial (Trial registration: ISRCTN45013173). As part of the trial, the STH prevalence in 8 randomly selected schools in the Kagera region receiving the Mikono Safi intervention will be compared to 8 schools in a randomly selected control arm.

While school-based WASH interventions in low- and middle-income countries have been examined with regards to a wide range of health and educational outcomes, including: diarrhea, respiratory infection, school attendance and health outcomes in the domestic environment [9-13], these studies have shown that the impact on such outcomes is inconsent . For the most part, this variability has been viewed through the lens of *intervention fidelity* and *compliance* – the extent to which schools and/or implementing organizations have provided WASH services in schools routinely and in a manner consistent with intervention protocols [14, 15] . However, for school-based hygiene interventions to result in health or educational impacts, they must also successfully change students' hygiene behaviours. Focusing on the implementation and delivery of school-based interventions alone has the potential to ignore the more complex relationships between an intervention and the proximal determinants of individual pupil HWWS behaviours. While studies have documented or tested the extent to which school-based interventions have resulted in changes in specific hygiene behaviours [16], more information is needed on the mechanisms through which interventions change behaviours.

For peer review only - http://bmjopen.bmj.com/site/about/guidelines.xhtml

The goal of our study was to explore the mechanisms by which and the extent to which the Mikono Safi intervention influenced the determinants of children's handwashing behaviour. Our primary research question was: to what extent has the Mikono Safi intervention influenced the capability, opportunity, and motiviation for HWWS among pupils attending intervention schools? Findings from this research will be used to modify current intervention approaches, to further understand the extent to which school-based interventions influence individual drivers of behaviour, and to provide contextual information for interpreting forthcoming trial results.

The Mikono Safi Intervention

The Mikono Safi intervention was informed by the Behaviour Centred Design process [17]] and combined a number of evidence-based components to foster and trigger handwashing behaviours at schools. These comprised: (i) class-room based teacher-led health education on the negative health effects and the transmission route of faeco-orally transmitted STHs, the importance of handwashing with water and soap at key times [18, 19]; (ii) triggering emotional drivers such as disgust, fear and nurture with regards to hygiene; (iii) demonstration of correct handwashing procedures; (iv) parental engagement through meetings at which parents were individually informed of their children's infection status; (v) provision of improved hand-washing stations with water and soap near school toilets; and (vi) application of environmental nudges to sub-conscioulsy trigger hygiene behaviour [16, 20]. The latter consisted of marked paths connecting toilets with hand-wash stations and painted hands on the hand-wash devises (Figure 1). Health education in class comprised 3 sessions of about 2 hours each, making use of games , posters and sets of comic-type

For peer review only - http://bmjopen.bmj.com/site/about/guidelines.xhtml

pictures of 2 students: Koku, a young girl who both washed hands at key times and supported others to adopt good hygiene behaviour, and Muta, a young boy who learned about appropriate hygiene behaviour in the process.

Figure 1 to be inserted here

Methods

Theoretical Framework

There are multiple models and frameworks that have been proposed to understand WASH behaviours [17, 21-23] . For our study we chose the COM-B framework [24] to inform study design, data collection, and analysis. COMB-B provides a parsimonious set of factors that determine behavioural outcomes: capability, opportunity, and motivation. .

Capability refers to an individual's psychological and physical capacity to complete HWWS. For the purposes of this study, key aspects of capability include: handwashing skills, knowledge related to HWWS, self-efficacy and proper social and educational support. Opportunity refers to factors external to the individual that make the behaviour possible or prompt it. For this study, we define opportunity as consistent access to soap and water for handwashing at a location that is convenient for children. Motivation is defined as the mental processes that direct behaviour. For the purposes of our study, we include cognitive, rational thought processes related to HWWS; habits; and emotional or motiviational repsonses as part of individual motivation.

For peer review only - http://bmjopen.bmj.com/site/about/guidelines.xhtml

For peer review only

Study Design, Sample and Data Collection Methods

This was a qualitative study conducted in 4 intervention schools involving students and teachers. We completed a range of qualitative data collection activities with students and teachers at 4 schools receiving the Mikono Safi intervention over a 6 month period. Purposive sampling was used to select both the schools and the study participants. Selection of schools ensured inclusion of rural and urban schools, and schools with both high and low pre-trial STH infection prevalence. Selection of teachers ensured inclusion of school administration, teachers in-charge of sanitation and other teachers who were involved in implementing hand hygiene education. Selection of students ensured inclusion of boys and girls, both younger and older children. In each school, we conducted two rounds of data collection. The first round of data was collected in April 2018; two months after the the intervention had been launched in the 4 schools. The second round was collected in the last week of October 2018; (Table 1). The purpose of conducting 2 rounds of data collection six months apart was to assess if the reported changes in behaviour and identified barriers and facilitators to proper HWWS changed over time.

February 2018	Intervention implementation
April 2018	Data collection, Round 1<br>- 2 focus group discussions completed at each participating school<br>- 4 in-depth interviews with teachers completed at each participating school
October 2018	Data collection, Round 2<br>- 5 friendship pair interviews completed at each participating school<br>- 4 in-depth interviews with teachers completed at each participating school

Table 1: Summary of data collection activities

For peer review only - http://bmjopen.bmj.com/site/about/guidelines.xhtml

In the first round of data collection, we completed 2 focus group discussions (FDGs) in each school, resulting in a total of 8 FGDs with a total of 60 students. The FGDs were age and gender segregated – resulting in 2 FGDs each for older boys, older girls, younger boys, and younger girls. During the FGDs, weexplored students' shared perceptions about the influence of the intervention on handwashing. We also assessed students' handwashing skills: at the end of each FGD, the field researcher walked with the participating students to the handwashing stations for students to demonstrate how they normally washed hands. The field researcher observed whether students washed both hands with soap. At each school, we conducted 4 in-depth interviews (IDIs) with teachers to collect information on their perceptions about students' knowledge regarding the relationship between HWWS and STH infection; experience with implementing handwashing lessons; availability and accessibility of handwashing materials at school, and perceived effectiveness of the intervention in targeting motivational and emotional drivers for handwashing.

For the second round of data collection (October 2018), we completed 5 friendship pair interviews (FPIs) at each school, for a total of 10 respondents per school. FPI is a method used to collect data from children where the target respondent is encouraged to come to the interview with a friend in order to create a conducive, non-threatening environment for the children[25] . The research team, with support from respective class teachers, solicited student volunteers. In each class, one student was selected. The selected student was asked to request his/her best friend to join the interview. Both the FGDs and FPIs collected information on students' knowledge regarding the relationship between HWW and STH infection, perceptions about implementation of handwashing lessons (including their perception about how consistent was the delivery of the handwashing messages),

For peer review only - http://bmjopen.bmj.com/site/about/guidelines.xhtml

availability and accessibility of handwashing materials at school, and connections between education materials and targeted motivational and emotional drivers of HWWS. Whereas FGDs were used to gain insights into children's shared understanding of how the intervention components had influenced handwashing and the ways in which individuals influenced by others in a group situation, we used FPIs to gain insight into individual children's practices and personal experiences. During the second round of data collection, we also completed semi-structured interviews with 4 teachers per school, these were the same teachers interviewed in round one.

Data was collected using pilot tested interview guide (supplementary file) . All interviews and FGDs were conducted in private empty rooms provided by the school administration. Each FGD/FPI lasted between 1 hour 30 minutes and 2 hours while interviews with teachers lasted between 30 and 45 minutes. All the interviews were collected in Kiswahili, transcribed, and translated in English by a bilingual speaker for analysis.

Research team

The data collection team comprised three Tanzanian social scientists at PhD or MSc level (EO; KM, OM) and two research assistants, 1 male and 1 female with experience in qualitative research methods. Prior to starting fieldwork the research assistants received additional one week's training in qualitative research methods. The first author (EO), participated in actual data collection at the beginning of each data collection round and coordinated the study. Before each round of data collection the study team visited the schools, explained the purpose of the study, discussed study activities with the school administration and made appointments for interviews and FGDs.

For peer review only - http://bmjopen.bmj.com/site/about/guidelines.xhtml

Public involvement

This qualitative study is assessing an intervention that was preceded by rigorous formative research. During the formative research there was extensive involvement of students, teachers and regional and district education officers and school WASH coordinators in developing, testing and modifying the various components of the intervention. The feedback received from these stakeholders informed the design and implementation of the intervention. Similarly, the interview and FGD guides used in the qualitative study were pilot-tested and revised to include feedback from the participants. After the end of the research, the findings from the main trial and from this qualitative study will be disseminated through stakeholders meetings.

For peer review only

Data analysis

Audio recorded interviews and FGDs were transcribed in Kiswahili and translated into English by the lead author and combined with field notes ready for analysis. Data were analyzed using Atlas.ti version 7, a qualitative data analysis and research software

The COM-B conceptual framework served as a guide for data analysis and was used to define themes in analysis. A thematic analysis approach was used to facilitate the structuring, description and interpretation of results [26] . During analysis, the first author read all interview and FGD transcripts and formulated draft codes based on recurring accounts and descriptions that were identified in the transcripts. The first and last authors

For peer review only - http://bmjopen.bmj.com/site/about/guidelines.xhtml

reviewed, discussed, and refined these codes and then mapped each code back to the COM-B framework. Each code was further classified into facilitators of and barriers to HWWS.

Data from both rounds were analyzed together using the same methods. Data from each of the two rounds of data collection were then directly compared [26].

Ethical considerations

Protocol, tools, and consent forms were reviewed by the National Health Research Ethics Review Committee of Tanzania (approval number: **NIMR/HQ/R.8a/Vol.IX/2665**) and the Ethics Committee of the London School of Hygiene and Tropical Medicine (**LSHTM Ethics Ref: 14539**). All participants provided informed assent (students) and consent (teachers); and parental consent was obtained for all participating students. Permission to interview students was sought from parents/guardians, while permission to interview teachers and school staff was sought from the school authorities. Personal data was anonymised, using ID numbers and stored data was stripped of names, and password protected for use by named research staff only.

For peer review only

Results

A total of 60 students participated in 8 FGDs and another group of 40 students participated in 20 FPIs . In addition, 32 in-depth interviews were held with 16 teachers all of whom were interviewed twice (Table 2) . All teachers and students selected for participation consented and took part.

For peer review only - http://bmjopen.bmj.com/site/about/guidelines.xhtml

Data collection round	Study participants					
	Students			Teachers		
	Male	Female	Age range	Male	Female	Age range
Round 1	30	30	7-15 years	7	9	23-52 years
Round 2	20	20	8-14 years	7	9	23-52 years

Table 2: Gender and age range of study participants

Factors that study participants identified as facilitators of good hand-washing behaviour were classified as according to the COM-B conceptual framework. Factors related to *capability* comprised increased knowledge and understanding of the relationship between handwashing and STH, knowledge of correct handwashing; practical skills regarding handwashing following practical demonstrations of handwashing. Factors related to *social and physical opportunity* included the successful development of a school community of students and teachers who supported and encouraged handwashing; and the improved quality, number and location of handwashing stations with access to both water and soap. Reflective and automatic factors related to increased *motivation* for handwashing comprised an increased understanding of the link between handwashing and good health that encouragds positive feeling about hand washing, disgust caused by realizing that STH infection is linked to ingesting faecal matter; the use of relatable child characters that triggered a feeling of nurture. The main barrier identified was the inconsistent availability of water and soap.

Capability

Overall, the Mikono Safi intervention had a positive effect on students' reported capability for HWWS. Two significant themes emerged: perceived improvements in handwashing skills

For peer review only - http://bmjopen.bmj.com/site/about/guidelines.xhtml

and techniques and reported improvements in knowledge about when and how to wash hands.

Handwashing skills: During handwashing demonstrations, all volunteers were able to follow correct handwashing steps. Participants reported to have mastered handwashing because they had participated in practical handwashing sessions:

> *The teacher took us to the handwashing stations and showed us how to wash hands. We were told that when you come from the toilet you wet your hands, apply soap, then scrub* **(FGD older boys, school 2).**

Both teachers and students believed that the acquired skills were important in facilitating handwashing.

Handwashing lessons and educational messaging: Both children and teachers indicated that teaching about handwashing and the use of educational messaging had greatly improved children's knowledge about how and when to wash hands. All children in both the FGDs and FPIs recalled receiving at least two lessons specific to handwashing in addition to the general sanitation and hygiene education provided within their normal school curriculum. Students understood the link between hand hygiene and infections. They also knew the key times at which they were required to wash hands.

> *Before the project came we had some tippy taps near the toilets. These were small and you had to step on them for water to pour on your hands. We did not always wash hands after using the toilet. But now were are motivated because we have been educated on the importance of handwashing so we now wash our hands always* **(FPI younger girls school, 1)**

For peer review only - http://bmjopen.bmj.com/site/about/guidelines.xhtml

Opportunity

The Mikono Safi intervention influenced opportunity for handwashing in two impotant ways - changing the physical opportunity for handwashing and altering the social environment related to handwashing at schools.

Physical Opportunity: Teachers and students alike indicated that availability and location of the handwashing facilities were important facilitators of handwashing. Teachers in particular noted that every child had to pass handwashing stations on their way to and from the latrine. Handwashing stations were visible and difficult to ignore:

> *Given the location of the handwashing stations… on the way out of the toilet, everybody who uses the toilet can see them on their way out…but you know children like to play with water, so making water and soap available for children makes handwashing part of a game (IDI,* **Teacher 2, school 3** *)*

> *…We wash our hands at the handwashing stations near the toilet. Before they installed the handwashing tanks we never used to wash hands because we did not have water to wash hands but these days we wash our hands because they have put the tanks. But in the past we never used to wash hands (***FPI, younger girls, school 3***)*

Children in the FGDs indicated that the improved handwashing facilities were not only easier and more convenient to use when compared to the tippy-taps that the school was using before but also the tanks contained much more water allowing a larger number of students to have access to water for washing hands.

> *"…Before the installation of the handwashing tanks we were using tippy-taps to wash hands and we had only few of them, the water would get finished quickly. But*

For peer review only - http://bmjopen.bmj.com/site/about/guidelines.xhtml

they were even difficult to use. With the current handwashing tanks you go there and open the tap and wash hands. The tanks are big and many students are able to wash hands using water in the tanks. ***(*FGD, older boys, school 1*)***

Beyond the physical availability of handwashing facilities, the most important facilitator to handwashing was the consistent provision of supplies (water and soap). In some schools, especially schools with a large student population, it was not uncommon for water to get used up early in the day - particularly during break time when the majority of the children used the toilet. Schools were able to mitigate these interruptions in water availability in various ways including creating rosters for fetching water. At chools that had well supervised and well implemented class rosters for fetching water more children indicated that water was consistently available at the handwashing station.

*…there are times when water gets finished, but in our school we have a teacher and students responsible for health and hygiene. So when water is finished from the tanks we inform the teacher or student leader and they send a group of students to bring water (***FPI, older girls, school 2)**

Water availability, however, was often determined by factors outside of the school's direct control. All schools experienced disruptions in water supply to varying degrees. In three of the four schools in the study, this was a problem that occurred during the morning break when the majority of the students used the toilets and during the dry season in which the majority of schools in the region experience water shortages. In one school this problem occurred multiple times during a week. During periods of limited availability, teachers needed to interrupt classes for certain groups of students in order for them to fill the tanks. Often, teachers were unwilling to interrupt classes, especially in schools that did not have a

For peer review only - http://bmjopen.bmj.com/site/about/guidelines.xhtml

well-established class roster for refilling water tanks. Lack of water had a negative influence on handwashing behaviours in general, particularly if water was unavailable for more than one day in a week.

In general, both students and teachers reported that soap was not consistently provided for washing hands. This was primarily an issue of distribution rather than of availability. At one school, for example, there was no soap available for students at the handwashing station when FGD participants were asked to demonstrate behaviours. Students noted that they had reported this to the teacher responsible earlier but in vain. However, when the moderator sent a student to the head teacher's office, the soap was provided. Similarly, discussions with school administrators indicated that, when provision of soap was inconsistent, it was usually available at the store. :

> *"There are a number of times during the week when soap is not available at the handwashing station. This can happen like three times in a week. If the soap is not at the handwashing station we usually go to the Head teacher's office to get the soap"* (**FGD, younger girls, school 3**)

> *There are times when the soap is not available because the teacher responsible did not check or the students have not reported. This happens sometimes but that usually is for a few hours in a day, not the whole day* (IDI, **Teacher 1, school 2)**

Social opportunity: Data suggest that the Mikono Safi intervention had created school communities that valued handwashing and that teachers were willing to support

For peer review only - http://bmjopen.bmj.com/site/about/guidelines.xhtml

handwashing activities through teaching and reminding children to wash hands, supervising children to fill the water tanks, and ensuring that soap was available. Students were also willing to participate in activities that ensured water and soap were available at the handwashing stations:

*"If I came out of the toilet and there is no water I will pick a bucket get water from the main reserve tank and fill the handwashing tanks so that other children and I can wash our hands" (***FGD, Older girls, school 2***)*

"Today I went to the toilet in the morning but there was no soap at the handwashing station at that time. I reported to the student in charge of sanitation and s/he promised to inform the teacher that there was not soap at the handwashing station"

*(***FPI, older girls, school 1***)*

Students, especially the youngest ones, were keen to report colleagues that did not wash hands after using the toilet. Teachers described experiences where students would report to them other students who did not wash hands.

*"Certainly, there are changes in children's handwashing behaviour. I am a teacher for the pre-primary class (the youngest group in school). What I have observed is that these days these young ones report colleagues that do not wash hands after using the toilet. If one of them used a toilet and did not wash hands the others will come running to you to report him or her; they will come and tell you 'teacher, so and so did not wash his/her hands after using the toilet'; they give you such reports (***IDI, teacher, school 2***)*

For peer review only - http://bmjopen.bmj.com/site/about/guidelines.xhtml

Motivation

Automatic motivation/Emotional triggers: Data suggest that the intervention was successful in reaching targeted emotional drivers of disgust, fear, and nuture in children. In particular, many children described Muta – the boy character depicted in the educational materials as a boy who did not know how to wash his hands - as repulsive and disgusting. On the other hand children found Koku (portrayed as a hygiene loving child) attractive and every child interviewed indicated that he or she would like be like Koku. Koku was described as clean and smart, but also kind and willing to teach a fellow student who may not have knowledge of handwashing yet.

> *I feel bad about Muta's behaviour; the fact that he did not wash his hand makes me feel disgusted…. but we like Koku because she was clean and she knew how to wash hands in order to protect herself from disease. Everyone would like to be like Koku, she is clean, smart and she likes helping others* (**FGD, older girls, school 4**)

> *… the teachers told us that if you use the toilet but you do not wash your hands and you go and eat anything you may be eating feces. So when you think about that you remember to wash your hands and you will remind all your friends to wash hands (**FGD, older girls, school 1**)*

Reflective motivation: Although introducing class rosters for fetching water was not part of the original intervention package, two of the four schools used rosters to ensure water was available at the handwashing facilities. For some schools this activity was led by students lead and for others by a teacher. For example, in schools that had active students' health

For peer review only - http://bmjopen.bmj.com/site/about/guidelines.xhtml

clubs, members of these clubs were charged with the responsibility for supervising all health related activities in school. Hence, schools which had active student health clubs developed the roster for health activities for each day of the week.

Students in schools that had student leaders involved in taking decisions about replenishing water and soap were more motivated to take action to ensure they have materials to wash hand. When children believed that they had power to influence their own handwashing practices –- they were willing to take an active role in activities that would facilitate handwashing among themselves and others.

> *"The students have received the handwashing lessons very well. They wash hand but they also encourage others to wash hands. For example if they saw a colleague who is dirty they encourage him/her to wash hand in order to avoid being labeled Muta, the character depicted in some of the education materials of a dirty boy…"* (**IDI, teacher 1, school 3**)

Changes over time: Results from the two rounds of data collection were compared to detect possible differences in terms of perceived barriers and facilitators related to capability, opportunity and motivation for handwashing. We did not find any differences. In both round one and two, students demonstrated good knowledge and skills for handwashing. Similarly, the opportunity for handwashing remained largely unchanged between the data collection points at round one and two. The installed handwashing facilities remained well maintained in all four schools. The schools continued to experience disruptions in water supply, and as before success in mitigating such disruptions varied from school to school.

For peer review only - http://bmjopen.bmj.com/site/about/guidelines.xhtml

Discussion

Our study set out to describe the barriers and facilitators to handwashing behaviour from the perspectives of teachers and students participating in the Mikono Safi intervention trial. Our results show that participants associated the intervention with improved skills and knowledge. Children's description of the intervention matched with the targeted emotional drivers of behaviours. Both students and teachers described changes in the social environment of the school that fostered better HWWS behaviours. However, the physical opportunity for handwashing was mixed. When both water and soap were available, the new handwashing stations increased the ease and convenience of handwashing among students. On the other hand, we noted that schools reported on-going challenges with water availability and that the provision of soap at handwashing stations – even when available at the school – remained inconsistent. Table 3 below summarises capability, opportunity, and motivational determinants of HWWS based associated with the Mokono Safi Intervention based on student and teacher reported data.

For peer review only - http://bmjopen.bmj.com/site/about/guidelines.xhtml

Determinant		Facilitators	Barriers
Capability	Psychological	Knowledge about the relationship between HWWS and STH<br><br>Knowledge of key moments for hand hygiene	
	Physical	Skills developed through HWWS demonstration and rehearsal	
Opportunity	Social	Teachers support and encourage HWWS<br><br>Peers who support HWWS	
	Physical	Improved quality, quantity, and location of HW stations	Inconsistent availability of water<br><br>Inconsistent availability of soap at HW stations
Motivation	Automatic	Emotional response (disgust / fear) triggered through messages and content<br><br>Relatable characters inspiring feelings of nurture among older students<br><br>Reminders (nudges) that reinforced handwashing habits	
	Reflective	Proactive student engagement in water collection and soap provision	

Table 3: Capability, Opportunity, and Motivational Determinants of HWWS as reported by the participants

For peer review only

For peer review only - http://bmjopen.bmj.com/site/about/guidelines.xhtml

All the students interviewed indicated knowing when, how and why one should wash hands. Although insufficient on its own, knowledge may be a necessary precursor for HWWS [27]. It is important to note that education and health messaging included in the Mikono Safi intervention were not abstract – they were focused around the risk of preventing intestinal worms. Studies in India have shown that providing individuals with specific- and actionable health information can improve WASH behaviours [27]. Having lessons that focus on hand hygiene and its risk with specific health outcomes in addition to regular sanitation and hygiene education may be a promising option for facilitating handwashing behaviour among school children.

Our education package also had an impact on the emotional drivers of hygiene behaviours. Posters and information went beyond just health messaging and included elements of disgust fear and nurture. Data suggest that we were successful in triggering emotional responses in children through our information campaigns. Studies have shown how motivational drivers can be successful in changing hygiene behaviours [28], although only a limited number of studies have explored handwashing motives related to children. The SuperAmma study in India [28] demonstrated significant improvement in hygiene behaviours based on messaging that targeted motivational drivers in isolation from health education. Our study shows how these emotional drivers can be combined with targeted health education to influence both motivation and capability.

Our intervention specifically targeted three emotional drivers: nurture, fear and disgust. However, nurture was seemingly the most important emotional driver that we were successful in targeting with intervention activities. Students spoke fondly of the fact that Koku (the female student appearing in education materials) helped her friends out; and

For peer review only - http://bmjopen.bmj.com/site/about/guidelines.xhtml

both teachers and students discussed the ways that the school had provided a supportive environment for handwashing. The salience of the nuture motive in our study population is consistent with other studies of student handwashing. For example, a study in Bangladesh that used covert video cameras to record handwashing behaviour found numerous instances of older children helping younger children washing hands and modelling positive behaviours for one another [29]. Students in our study responded positively to the nurture messages, suggesting that positive peer pressures are a potential avenue for improving HWWS in schools.

Making handwashing facilities visible, difficult to ignore but also convenient, were among the attributes mentioned as important by both teachers and students. However, beyond the physical positioning and convenience, the most important facilitator to handwashing was consistent provision of water and soap. Although all schools experienced disruptions in water supply especially at peak hours, some schools that were able to implement a well supervised class rosters for fetching water succeeded to avoid interruptions in water availability at handwashing stations. Similarly students and teachers suggested that inconsistency in the availability of soap at the handwashing stations was largely an issue of internal distribution rather than of external logistics. Our data suggests that while the Mikono Safi intervention was successful in changing capability and motivation, the opportunity for HWWS may not have been sufficiently improved in some schools, either due to external factors such as the dry season, or to internal factors such as the lack of class duty rosters for the collection of water or soap.

Our study has some limitations : firstly, this was a qualitative study involving a small sample of schools, teachers and pupils, and therefore our results may not be generalizable to other

For peer review only - http://bmjopen.bmj.com/site/about/guidelines.xhtml

intervention schools. Secondly, findings are primarily based on individual interviews and FGDs. Data from direct observation of students' behaviour was not collected during the qualitative study. However, FGDs, FPIs with students and IDIs with teachers proved to be well suited methods to examine barriers and facilitators to appropriate handwashing behaviour, and produced consistent results. Similarly, this study was conducted in Tanzania and the results may not be generalizable to other countries. We also do not have information on drivers of behaviour before the intervention was implemented. Students and teachers were aware of our affiliation with the larger Mikono Safi intervention trial and courtesy bias may have influenced both teachers and students to provide answers they assumed we wanted to hear. Reports on changes in behaviour should therefore be interpreted with caution. However, the majority of our study explored the drivers of behaviour rather than the behaviours themselves.

The COM-B Framework provided a prudent way to explore the success of the Mikono Safi intervention in addressing the barriers and facilitators for handwashing in our study population. Our analysis was based on drivers and facilitators among students. Viewed from an instititutional perspective, several of our key findings could relate to other COM-B determinants. For example, consistent provision of soap from the institution's perspective may be a factor of reflective motivation – schools may not have prioritized soap distribution or they could have made conscious decisions to without materials from students out of budget or supply concerns. Given the important role in intervention compliance in several school-based WASH interventions[11, 14], more theoretically informed research is needed to unpack the drivers of institutional factors that contribute to behavioural outcomes.

For peer review only - http://bmjopen.bmj.com/site/about/guidelines.xhtml

In conclusion, the Mikono Safi intervention was associated with improved motivation and capability for handwashing, but ensuring the opportunity for handwashing remained a challenge at some schools, especially with respect to a consistent availability of water. For such schools a much stronger multi-sectoral collaboration between the departments for education and water may be required. Results from the forthcoming trial will allow us to understand the impact this intervention had on child health outcomes. Future research focused on hygiene and hygiene behaviour change should further explore the ways in which motivational and educational messaging can change specific determinants of behaviour; new intervention modalities that promote school-level adherence to routine provision of basic supplies need development and testing in resource-scarce environments like Tanzania

For peer review only

For peer review only - http://bmjopen.bmj.com/site/about/guidelines.xhtml

References

1. WHO *Soil-transmitted helminth infections updates, in facts sheet*. 2019. https://www.who.int/news-room/fact-sheets/detail/soil-transmitted-helminth-infections.
2. Drake, L.J., et al., *Geohelminth infections (ascariasis, trichuriasis, and hookworm): Cognitive and developmental impacts.* Seminars in Pediatric Infectious Diseases, 2000. **11**(4): p. 245-251.
3. Hotez, P.J., et al., *Hookworm Infection.* New England Journal of Medicine, 2004. **351**(8): p. 799-807.
4. WHO, *Preventive chemotherapy in human helminthiasis. Coordinated use of antihelminthic drugs in control interventions: a manual for health professionals and programme managers*. 2006, World Health Organization: Geneva.
5. MOHSW, *Tanzania National Master Plan for Neglected Tropical Diseases*. 2009, Tanzania Ministry of Health and Social Welfare.
6. GAHI, *Distribution of soil-transmitted helminth survey data in Tanzania*. 2017, Global Atlas of helminth infections.
7. WHO *Soil-transmitted helminth infections, in Fact sheet* 2013b. https://www.who.int/news-room/fact-sheets/detail/soil-transmitted-helminth-infections.
8. Yap, P., et al., *Rapid Re-Infection with Soil-Transmitted Helminths after Triple-Dose Albendazole Treatment of School-Aged Children in Yunnan, People's Republic of China.* The American journal of tropical medicine and hygiene, 2013. **89**.
9. Caruso, B.A., et al., *Assessing the impact of a school-based latrine cleaning and handwashing program on pupil absence in Nyanza Province, Kenya: a cluster-randomized trial.* Tropical Medicine & International Health, 2014. **19**(10): p. 1185-1197.
10. Chard, A.N., et al., *The impact of school water, sanitation, and hygiene improvements on infectious disease using serum antibody detection.* PLOS Neglected Tropical Diseases, 2018. **12**(4): p. e0006418.
11. Dreibelbis, R., et al., *The Impact of School Water, Sanitation, and Hygiene Interventions on the Health of Younger Siblings of Pupils: a Cluster-Randomized Trial in Kenya.* American Journal of Public Health, 2013. **104**(1): p. e91-e97.
12. Freeman, M., et al., *The impact of improved school water, sanitation and hygiene access on pupil diarrhea: A cluster randomized trial.* Am J Trop Med Hyg, 2012. **87**(Supplement 1): p. 428.
13. Freeman, M.C., et al., *Systematic review: Hygiene and health: systematic review of handwashing practices worldwide and update of health effects.* Tropical Medicine & International Health, 2014. **19**(8): p. 906-916.
14. Chard, A.N. and M.C. Freeman, *Design, Intervention Fidelity, and Behavioral Outcomes of a School-Based Water, Sanitation, and Hygiene Cluster-Randomized Trial in Laos.* International Journal of Environmental Research and Public Health, 2018. **15**(4): p. 570.
15. Garn, J., et al., *The Role of Adherence on the Impact of a School-Based Water, Sanitation, and Hygiene Intervention in Mali.* The American Journal of Tropical Medicine and Hygiene, 2017. **96**: p. 16-0558.
16. Dreibelbis, R., et al., *Behavior Change without Behavior Change Communication: Nudging Handwashing among Primary School Students in Bangladesh.* International journal of environmental research and public health, 2016. **13**(1): p. 129.
17. Aunger, R. and V. Curtis, *Behaviour Centred Design: towards an applied science of behaviour change.* Health psychology review, 2016. **10**: p. 1-22.
18. Kapp, K., *The gamification of learning and instruction: game-based methods and strategies for training and education*. 2012: John Wiley & Sons.

For peer review only - http://bmjopen.bmj.com/site/about/guidelines.xhtml

19. Watson, J., et al., *Child's play: Harnessing play and curiosity motives to improve child handwashing in a humanitarian setting.* International Journal of Hygiene and Environmental Health, 2019. **222**(2): p. 177-182.
20. Grover, E., et al., *Comparing the behavioural impact of a nudge-based handwashing intervention to high-intensity hygiene education: a cluster-randomised trial in rural Bangladesh.* Tropical Medicine & International Health, 2018. **23**(1): p. 10-25.
21. Coombes, Y. and J. Devine, *Introducing FOAM: A Framework to Analyze Handwashing Behaviors to Design Effective Handwashing Programmes* 2010, World Bank: Washington, DC.
22. Dreibelbis, R., et al., *The Integrated Behavioural Model for Water, Sanitation, and Hygiene: a systematic review of behavioural models and a framework for designing and evaluating behaviour change interventions in infrastructure-restricted settings.* BMC Public Health, 2013. **13**(1): p. 1015.
23. Mosler, H.-J., *A systematic approach to behavior change interventions for the water and sanitation sector in developing countries: a conceptual model, a review, and a guideline.* International Journal of Environmental Health Research, 2012. **22**(5): p. 431-449.
24. Michie, S., M.M. van Stralen, and R. West, *The behaviour change wheel: A new method for characterising and designing behaviour change interventions.* Implementation Science, 2011. **6**(1): p. 42.
25. Wilson, A., A. Onwuegbuzie, and L.P. Manning, *Using paired depth interviews to collect qualitative data.* The Qualitative Report, 2016. **21**(9): p. 1549-1573.
26. Attride-Stirling, J., *Thematic networks: an analytic tool for qualitative research.* Qualitative Research, 2001. **1**(3): p. 385-405.
27. Trent, M., et al., *Access to Household Water Quality Information Leads to Safer Water: A Cluster Randomized Controlled Trial in india.* Environmental Science & Technology, 2018. **52**(9): p. 5319-5329.
28. Biran, A., et al., *Effect of a behaviour-change intervention on handwashing with soap in India (SuperAmma): a cluster-randomised trial.* The Lancet Global Health, 2014. **2**(3): p. e145-e154.
29. Grover, E., et al., *Social Influence on Handwashing with Soap: Results from a Cluster Randomized Controlled Trial in Bangladesh.* The American Journal of Tropical Medicine and Hygiene, 2018. **99**.

For peer review only

Footnotes

Acknowledgments: We thank John Robert and Winnie Muriba for assisting with data collection and transcription of the interviews and focus group discussions. We are grateful to the parents for allowing their children to take part in the study and the school administration of the 4 schools for giving us permission to talk to students and teachers. We are grateful to the participating students and teachers for their time and contributions to the study. This study was funded by the Department of International Development (DFID) of the United Kingdom (UK) through the SHARE Consortium programme, based at the London School of Hygiene and Tropical Medicine (LSHTM).

Contributors: EO conceived the study and was the investigator with overall responsibility for the management of the study. EO and RD were responsible for the original design and protocol. SK, HG, SK, contributed to the study design methodology and obtaining funding. KM and OM supervised fieldwork and reviewed the draft manuscript. EO analyzed the data and wrote the first draft of the manuscript. RD, HG, SK, and SK provided a critical review for important intellectual content. All authors were involved in the final version of the manuscript and approved the manuscript for submission.

Disclaimer: The views expressed are those of the authors and not necessarily those of the funder

Competing interests statement: There are no competing interests for any authors

Data sharing statement: The data are anonymised transcripts and are available upon reasonable request by emailing elialilia@mitu.or.tz..

For peer review only - http://bmjopen.bmj.com/site/about/guidelines.xhtml

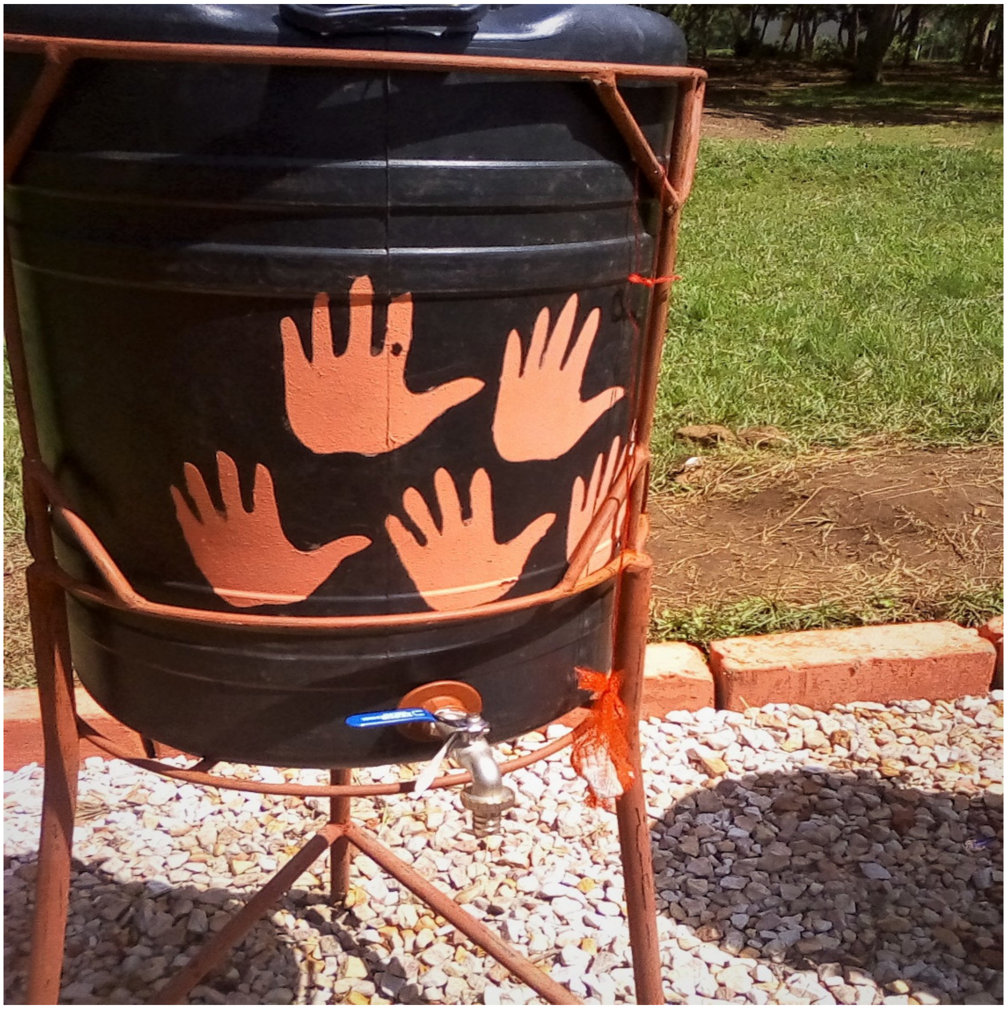

Figure 1: Handwashing facility with “nudges” installed in the intervention schools

STUDY GUIDES

Appendix 1: FGD/Friendship Pair Interview question guide for students

Name of District.. Name of School...
Age of the respondent............................ Class of the respondent..........................

A: Knowledge about HWWS and helminth infections

1. Tell me about the last time you washed your hands.
    a. What did you use?
    b. Where were you? (*School, home, friend's house, etc*.)
    c. What were you doing immediately before you washed your hands?
    d. What did you do after you washed your hands?
    e. Potential probes:
        i. Are there other times during the day you usually wash your hands?
        ii. Why is it important to wash hands then?
2. The last time you washed your hands; can you show me how you did it? (consider bringing soap and water to interview)
    a. Is that how you always wash your hands? [*Probe on any different times or ways that they describe].*
3. What happens if you don't wash your hands?[*Probe on responses]*
4. Imagine you are at school and you go to wash your hands.
    a. You find there is no soap at the handwashing station. What would you do? (*Probe on responses: Then what would you do? Would you try something else? Etc*.)
    b. Now imagine there is no water at the handwashing station. What would you do then? (Probe…)
5. Now, imagine you are at home and want to wash your hands. What do you do if there is no soap? No water?

B: Implementation of handwashing lessons

6. Please tell me about any lessons you've had in class recently about handwashing
7. What do you remember about the lesson? (Probe on as many specifics as possible.)
8. During the lesson,
    a. What did the teacher do?
    b. What did the children do?
9. What parts did you like most about the lesson? Why? What was your favourite part of the lessons?

For peer review only - http://bmjopen.bmj.com/site/about/guidelines.xhtml

C: Availability and accessibility of Handwashing materials

10. While in school, where do you go to wash your hands?
11. Are there times when you want to wash your hands but water is finished? If yes, tell me about the last time you went but the water was finished. (Probe on how long ago, what time of day, what they did in response.) How often does this happen?
12. Are there times when you need soap to wash your hands but soap is finished? Same as above.

D: Effectiveness of Mikono Safi in targeting in motivational and emotional drivers of handwashing with soap

13. During the handwashing exercise that we talked about earlier, teachers used some examples,
    a. What are the examples?
    b. Which of the examples do you remember most?
    c. Why do you remember that example?
14. [Bring and use some of the motivational messages during the interviews; Show the children the Muta and Koku flip chart then ask]
    a. Please tell me what you see on the flip chart
    b. What do you think about Muta?
    c. How does Muta's poor hygiene behavior make you feel?
    d. What do think about Koku?
    e. How does Koku's good hygiene make you feel?
    f. Imagine Muta was you young brother, what would to help him?
15. Show the children show the children the side by the side poster
    a) What do you see on the poster? [Probe on the response]
    b) Focusing on the side of the child with worms
        i. What is the girl/boy doing wrong?
        ii. How does the girl/boy's behavior make you feel?
        iii. What would you do if you were this girl/boy?
        iv. What would you help if this boy/girl was your little brother/sister?
16. If you saw child from standard one coming from the toilet and running to play, what would you do? Why?
17.

E: Consistence in message delivery

18. Which day of the week do you usually receive the hand washing lessons?
19. What time of the day do the teachers come for hand washing lessons?
20. What activities do children participate in during handwashing lessons?

For peer review only - http://bmjopen.bmj.com/site/about/guidelines.xhtml

Appendix 2: In-depth Interview guide for Teachers/school staff

Name of District....................................... Name of School..
Age of the respondent............................ Education level of the respondent.........................

A: Knowledge about HWWS and helminth infections

1. In your opinion, how much do children know about when and how to washing hands? [*probe on response*]
2. What do you think motivates them to wash hands?
3. What do you think discourages from washing hands at critical times?
4. In your observation, If for example, children want to wash hands but there is no water, what do they usually do?
5. In your observation, If for example, children want to wash hands but there is no water, what do they usually do?

B: Lessons implemented and appropriateness of the lessons

6. Please tell me how handwashing lessons are delivered in your school
   a. What has been your role in delivering these lessons?
   b. In what classes are the lessons delivered?
   c. How often are these lessons delivered?
   d. What activities are the children involved in during these lessons?
   e. What has been your experience in delivering these lessons?
7. How would you describe the involvement of teachers and school staff in delivering handwashing lessons in your school? [*probe on the answer*]
8. What motivates teachers to get involved in delivering handwashing lessons?
9. What discourages teachers from getting involved in delivering handwashing lessons
10. What is your opinion about the contents of the handwashing curriculum?
11. How far if the curriculum is able address knowledge and skill gaps related to handwashing in children?
12. Do you have any recommendations to improve the handwashing curriculum?

C: Availability and accessibility of Handwashing materials

13. Tell me about the availability of water and soap in your school
    a. How does the school ensure water is available for children to wash hands?
    b. How does the school ensure soap is available for children to wash hands?
14. Are there times when water is not available for children to wash hands? How often does this happen?
15. Are there times when soap is finished? How often does this happen?
16. How does the school maintain the handwashing stand?

For peer review only - http://bmjopen.bmj.com/site/about/guidelines.xhtml

17. How does your school ensure that latrines are kept clean? [*probe for who is involved*]
18. How does your school ensure there are sufficient and appropriate supplies for latrine cleaning?
19. How are latrines managed and maintained?[probe about who is involved]

D: Effectiveness of Mikono Safi in targeting in motivational and emotional drivers of handwashing with soap

20. How would you describe children response to handwashing lessons?
21. Among the motivational messages used in the handwashing curriculum, what parts do children enjoy most during handwashing lessons? Why?
22. In your view, motivational messages; what parts do children remember most about handwashing lessons? Why?
23. What changes have you observed in children's handwashing behavior?
    a. Is there a difference between girls and boys?
    b. Are there differences among age groups?

For peer review only

For peer review only - http://bmjopen.bmj.com/site/about/guidelines.xhtml

COREQ (COnsolidated criteria for REporting Qualitative research) Checklist

A checklist of items that should be included in reports of qualitative research. You must report the page number in your manuscript where you consider each of the items listed in this checklist. If you have not included this information, either revise your manuscript accordingly before submitting or note N/A.

Topic	Item No.	Guide Questions/Description	Reported on Page No.
**Domain 1: Research team and reflexivity**			
Personal characteristics			
Interviewer/facilitator	1	Which author/s conducted the interview or focus group?	9
Credentials	2	What were the researcher's credentials? E.g. PhD, MD	9
Occupation	3	What was their occupation at the time of the study?	9
Gender	4	Was the researcher male or female?	9
Experience and training	5	What experience or training did the researcher have?	9
Relationship with participants			
Relationship established	6	Was a relationship established prior to study commencement?	9
Participant knowledge of the interviewer	7	What did the participants know about the researcher? e.g. personal goals, reasons for doing the research	9
Interviewer characteristics	8	What characteristics were reported about the inter viewer/facilitator? e.g. Bias, assumptions, reasons and interests in the research topic	9
**Domain 2: Study design**			
Theoretical framework			
Methodological orientation and Theory	9	What methodological orientation was stated to underpin the study? e.g. grounded theory, discourse analysis, ethnography, phenomenology, content analysis	4, 9, 10
Participant selection			
Sampling	10	How were participants selected? e.g. purposive, convenience, consecutive, snowball	7
Method of approach	11	How were participants approached? e.g. face-to-face, telephone, mail, email	9
Sample size	12	How many participants were in the study?	7 & 10
Non-participation	13	How many people refused to participate or dropped out? Reasons?	9
Setting			
Setting of data collection	14	Where was the data collected? e.g. home, clinic, workplace	9
Presence of non-participants	15	Was anyone else present besides the participants and researchers?	9
Description of sample	16	What are the important characteristics of the sample? e.g. demographic data, date	10
Data collection			
Interview guide	17	Were questions, prompts, guides provided by the authors? Was it pilot tested?	9
Repeat interviews	18	Were repeat inter views carried out? If yes, how many?	8
Audio/visual recording	19	Did the research use audio or visual recording to collect the data?	9
Field notes	20	Were field notes made during and/or after the inter view or focus group?	9
Duration	21	What was the duration of the inter views or focus group?	9
Data saturation	22	Was data saturation discussed?	__
Transcripts returned	23	Were transcripts returned to participants for comment and/or	__

For peer review only - http://bmjopen.bmj.com/site/about/guidelines.xhtml

Topic	Item No.	Guide Questions/Description	Reported on Page No.
		correction?	
**Domain 3: analysis and findings**			
Data analysis			
Number of data coders	24	How many data coders coded the data?	10
Description of the coding tree	25	Did authors provide a description of the coding tree?	—
Derivation of themes	26	Were themes identified in advance or derived from the data?	10
Software	27	What software, if applicable, was used to manage the data?	9
Participant checking	28	Did participants provide feedback on the findings?	—
Reporting			
Quotations presented	29	Were participant quotations presented to illustrate the themes/findings? Was each quotation identified? e.g. participant number	11-18
Data and findings consistent	30	Was there consistency between the data presented and the findings?	11-18
Clarity of major themes	31	Were major themes clearly presented in the findings?	11-18
Clarity of minor themes	32	Is there a description of diverse cases or discussion of minor themes?	11-18

Developed from: Tong A, Sainsbury P, Craig J. Consolidated criteria for reporting qualitative research (COREQ): a 32-item checklist for interviews and focus groups. *International Journal for Quality in Health Care*. 2007. Volume 19, Number 6: pp. 349 – 357

Once you have completed this checklist, please save a copy and upload it as part of your submission. DO NOT include this checklist as part of the main manuscript document. It must be uploaded as a separate file.

For peer review only

BMJ Open

Factors perceived to facilitate or hinder handwashing among primary students: a qualitative assessment of the Mikono Safi intervention schools in NW Tanzania

Journal:	*BMJ Open*
Manuscript ID	bmjopen-2019-030947.R2
Article Type:	Original research
Date Submitted by the Author:	26-Oct-2019

Complete List of Authors:	Okello, Elialilia; Mwanza Intervention Trials Unit, MITU; National Institute for Medical Research (NIMR), Mwanza Centre<br>Kapiga, Saidi ; London School of Hygiene and Tropical Medicine, Infectious Disease Epidemiology; Mwanza Intervention Trials Unit<br>Grosskurth, Heiner; London School of Hygiene and Tropical Medicine, Epidemiology and Public Health; NIMR, Mwanza Centre<br>Makata, Kenneth; Mwanza Intervention Trials Unit, MITU; NIMR, Mwanza Centre<br>Mcharo, Onike; Mwanza Intervention Trials Unit, MITU; NIMR, Mwanza Centre<br>Kinungh'i, Safari; National Insitute for Medcial Research, Mwanza Centre<br>Dreibelbis , Robert; London School of Hygiene and Tropical Medicine

**Primary Subject Heading**:	Public health
Secondary Subject Heading:	Qualitative research
Keywords:	Handwashing, COM-B Model, QUALITATIVE RESEARCH

SCHOLARONE™
Manuscripts

Factors perceived to facilitate or hinder handwashing among primary students: a qualitative assessment of the Mikono Safi intervention schools in NW Tanzania

Authors:

1. Elialilia Okello*, Mwanza Intervention Trials Unit (MITU), P. O Box 11936, Mwanza & National Institute for Medical Research (NIMR), Mwanza Centre, Tanzania; E-mail addresses: elialilia.okello@mitu.or.tz; elly16.sp@gmail.com

2. Saidi Kapiga: Mwanza Intervention Trials Unit, Mwanza Tanzania & London School of Hygiene and Tropical Medicine (LSHTM), London UK & NIMR, Mwanza Centre, Tanzania

3. Heiner Grosskurth: Mwanza Intervention Trials Unit, Mwanza & London School of Hygiene and Tropical Medicine, London UK & NIMR, Mwanza Centre, Tanzania

4. Kenneth Makata: Mwanza Intervention Trials Unit, Mwanza & NIMR, Mwanza Centre, Tanzania

5. Onike Mcharo: Mwanza Intervention Trials Unit, Mwanza & NIMR, Mwanza Centre, Tanzania

6. Safari Kinungh'i: National Institute for Medical Research, Mwanza Centre, Tanzania

7. Robert Dreibelbis: London School of Hygiene and Tropical Medicine, London UK

****Corresponding Author***

For peer review only - http://bmjopen.bmj.com/site/about/guidelines.xhtml

Abstract

Objective: To qualitatively assess the effects of a multi-modal school-based water, sanitation, and hygiene (WASH) intervention on handwashing behaviour among primary students in NW Tanzania.

Design: The study was a qualitative assessment of barriers and facilitators to handwashing among students attending primary schools participating in the Mikono Safi Trial (Kiswahili for 'Clean Hands; trial registration: ISRCTN45013173), a cluster-randomized trial assessing the impact of a school-based WASH intervention on selected soil transmitted helminth infections. Data collection methods included in-depth interviews with teachers, focus group discussions and friendship pair interviews with students collected between April and October 2018. The Capability-Opportunity-Motivation and Behaviour (COM-B) model was used to inform data collection and analysis.

Setting: The study was conducted in 4 purposively selected intervention schools in three districts of Kagera region, North Western Tanzania (Bukoba urban, Bukoba rural and Muleba dsitricts).

Participants: Participants comprised 16 purposively selected teachers aged between 23 and 52 years and 100 students aged 7 to 15 years

Results: The Mikono Safi intervention increased students' reported capability and motivation to wash their hands with soap at key times, particularly after visiting the toilet.

For peer review only - http://bmjopen.bmj.com/site/about/guidelines.xhtml

Improvements in students' handwashing knowledge and skills were reported by both teachers and students, and motivation for hand washing was enhanced by emotional drivers such as disgust, fear and nurture. Newly established handwashing stations improved the physical opportunity to wash hands, although the availability of water and the provision of soap was not always consistent (e.g. due to internal organisational shortcomings or during the dry season). Students and teachers were actively engaged in intervention implementation which created a school community that valued and supported improved hand hygiene.

Conclusion: The intervention was successful in improving capability and motivation for handwashing. Handwashing opportunity was also greatly improved, although the supply with water and soap was sometimes interrupted, calling for much stronger multi-sectoral collaboration to improve access to water at schools.

***Key words**: Handwashing, COM B Model, qualitative research, primary school, hand hygiene*

For peer review only - http://bmjopen.bmj.com/site/about/guidelines.xhtml

For peer review only

Strengths and limitations of this study

- In-depth interviews of teachers and focus group discussions and indepth friendship pair interviews proved to be suitable methods to examine barriers to and facilitators of appropriate handwashing behaviour.
- These methods generated consistent results suggesting that the Mikono Safi intervention was effective in improving handwashing behaviour
- This was a qualitative study involving a small purposively selected sample of schools, teachers and pupils and may therefore not be representative for all 8 intervention schools participating in the trial or for other primary schools in Tanzania.

For peer review only

Introduction

Soil-transmitted helminth (STH) infections are among the most prevalent infections worldwide, affecting over 1.5 billion people [1] . These infections have demonstrable negative impacts on child development [2, 3] . Current control strategies focus on school-based mass drug administration (MDA) programmes. Since 2006 the World Health Organisation recommends annual deworming among all at-risk people living in endemic areas if the population prevalence of soil-transmitted helminth infections exceeds 20%, and twice a year if it exceeds 50%, using orally perscribed albendazole (400 mg) or mebendazole (500 mg) which are both deemed effective, inexpensive and easy to administer by non-medical personnel (e.g. teachers) [4] . In 2009, the Tanzanian government began school-based MDA for STH control. Kagera, a region situated in north-western Tanzania west of Lake Victoria, has been a major focus for this MDA initiative [5] . School children of this area have received annual treatment with albendazole for the past eight years. However, recent survey data on the distribution of STH in Tanzania shows that the prevalence of these infections has remained high [6] , which is in keeping with recent research showing rapid re-infection following MDA programmes when water, sanitation and hygiene (WASH) conditions remain poor [7] . A recent randomized controlled trial in China demonstrated that the promotion of handwashing with soap in schools can result in significant reductions in STH prevalence [8], suggesting that school-based WASH interventions could play a significant role in STH infection control.

The Mikono Safi (Swahili for “clean hands”) intervention was designed to increase handwashing with soap (HWWS) among school-aged children in an attempt to sustain the effects of routine MDA. It is a multi-modal intervention, combining health education, games

For peer review only - http://bmjopen.bmj.com/site/about/guidelines.xhtml

and stories targeting key motivational drivers, improvements in school handwashing infrastructure, and training and support to school staff. Training for teachers in all the intervention school was done by a qualified public health specialist and a social scientist with a Master's degree in sociology. Further details are provided below. The impact of the Mikono Safi intervention is being assessed in an ongoing cluster-randomized trial (Trial registration: ISRCTN45013173). As part of the trial, the STH prevalence in 8 randomly selected schools in the Kagera region receiving the Mikono Safi intervention will be compared to 8 schools in a randomly selected control arm.

While school-based WASH interventions in low- and middle-income countries have been examined with regards to a wide range of health and educational outcomes, including: diarrhea, respiratory infection, school attendance and health outcomes in the domestic environment [9-13], these studies have shown that the impact on such outcomes is inconsistent. For the most part, this variability has been viewed through the lens of *intervention fidelity* and *compliance* – the extent to which schools and/or implementing organizations have provided WASH services in schools routinely and in a manner consistent with intervention protocols [14, 15] . However, for school-based hygiene interventions to result in health or educational impacts, they must also successfully change students' hygiene behaviours. Focusing on the implementation and delivery of school-based interventions alone has the potential to ignore the more complex relationships between an intervention and the proximal determinants of individual pupil HWWS behaviours. While studies have documented or tested the extent to which school-based interventions have resulted in changes in specific hygiene behaviours [16], more information is needed on the mechanisms through which interventions change behaviours.

For peer review only - http://bmjopen.bmj.com/site/about/guidelines.xhtml

The goal of our study was to explore the mechanisms by which and the extent to which the Mikono Safi intervention influenced the determinants of children's handwashing behaviour. Our primary research question was: to what extent has the Mikono Safi intervention influenced the capability, opportunity, and motiviation for HWWS among pupils attending intervention schools? Findings from this research will be used to modify current intervention approaches, to further understand the extent to which school-based interventions influence individual drivers of behaviour, and to provide contextual information for interpreting forthcoming trial results.

The Mikono Safi Intervention

The Mikono Safi intervention was informed by the Behaviour Centred Design process [17] and combined a number of evidence-based components to foster and trigger handwashing behaviours at schools. These comprised: (i) class-room based teacher-led health education on the negative health effects and the transmission route of faeco-orally transmitted STHs, the importance of handwashing with water and soap at key times [18, 19]; (ii) messaging and activities designed to trigger emotional drivers of HWWS such as disgust, fear and nurture with regards to hygiene; (iii) demonstration of correct handwashing procedures; (iv) parental engagement through meetings at which parents were individually informed of their children's infection status; (v) provision of improved hand-washing stations with water and soap near school toilets; and (vi) application of environmental nudges to sub-consciouslsy trigger hygiene behaviour [16, 20]. The latter consisted of marked paths connecting toilets with hand-wash stations and painted hands on the hand-wash devises (Figure 1). Health education in class comprised 3 sessions of about 2 hours each, making use of games ,

For peer review only - http://bmjopen.bmj.com/site/about/guidelines.xhtml

posters and sets of comic-type pictures of 2 students: Koku, a young girl who both washed hands at key times and supported others to adopt good hygiene behaviour, and Muta, a young boy who learned about appropriate hygiene behaviour in the process. Prior to implementation in each school, the study team completed a half-day training at each participating study school with 8 to 10 teachers to train teachers on education and messaging activities, determine the schedule for school-based activities, and develop school-specific maintenance plans for handwashing facilities and materials. Full details on the Mikono Safi intervention are described in a forthcoming publication (Makata et al., in review)

Figure 1 to be inserted here

Methods

Theoretical Framework

There are multiple models and frameworks that have been proposed to understand WASH behaviours [17, 21-23]. For our study we chose the COM-B framework [24] to inform study design, data collection, and analysis. COM-B, relative to other behaviour change theories and models, provides an intuitive and flexible approach to understanding behaviours that is applicable across a number of behaviours and contexts. COM-B identifies three broad categories of determinants determine behavioural outcomes: capability, opportunity, and motivation. Our study was was focused on HWWS. As such, we refer to capability as an individual's psychological and physical capacity to complete HWWS. Key aspects of

For peer review only - http://bmjopen.bmj.com/site/about/guidelines.xhtml

capability include: handwashing skills, knowledge related to HWWS, and self-efficacy. We define opportunity as consistent access to soap and water for handwashing at a location that is convenient for children and proper social and education support. Motivation is defined as the mental processes that direct behaviour. For the purposes of our study, we include cognitive, rational thought processes related to HWWS; habits; and emotional or motiviational repsonses as part of individual motivation.

Study Design, Sample and Data Collection Methods

This was a qualitative study where we completed a range of qualitative data collection activities with students and teachers at 4 schools receiving the Mikono Safi intervention over a 6 month period.. We used purposive sampling to select schools reflecting the range of schools participating in the Mikono Safi Trial. This included selected 2 urban and 2 rural schools for participation in our qualitative assessment from the 4 urban and 4 rural schools participating in the Mikono Safi Trial. We purposively sampled schools with both high and low pre-trial STH infection prevalence. At the school-level, selection of teachers ensured inclusion of school administration, teachers in-charge of sanitation and other teachers who were involved in implementing hand hygiene education. Selection of students ensured inclusion of boys and girls, both younger and older children.

In each school, we completed two rounds of data collection. The first round of data was collected in April 2018; two months after the Mikono Safi intervention had been launched in the 4 schools. The second round was collected in the last week of October 2018; (Table 1). The purpose of conducting two rounds of data collection 6 months apart was to assess if the

For peer review only - http://bmjopen.bmj.com/site/about/guidelines.xhtml

reported changes in behaviour and identified barriers and facilitators to proper HWWS changed over time.

February 2018	Intervention implementation
April 2018	Data collection, Round 1<br>- 2 focus group discussions completed at each participating school<br>- 4 in-depth interviews with teachers completed at each participating school
October 2018	Data collection, Round 2<br>- 5 friendship pair interviews completed at each participating school<br>- 4 in-depth interviews with teachers completed at each participating school

Table 1: Summary of data collection activities

In the first round of data collection, we completed 2 focus group discussions (FDGs) in each school, resulting in a total of 8 FGDs with a total of 60 students. The number of FGD participants ranged between 7 and 10. The FGDs were age and gender segregated – resulting in 2 FGDs each for older boys, older girls, younger boys, and younger girls. During the FGDs, weexplored students' shared perceptions about the influence of the intervention on handwashing. We also assessed students' handwashing skills: at the end of each FGD, the field researcher walked with the participating students to the handwashing stations for students to demonstrate how they normally washed hands. Hand washing education sessions had a practical component that took students through 5 hand washing steps which included: i) wetting hands, ii) putting soaps, iii) scrubbing the palms together, iv) scrubbing between the fingers and nails while counting up to 10, and v) rinsing hands with running water. Handwashing skills for each student were assessed during observation by noting how many of the five recommended steps of handwashing were completed.

In addition to FGDs, we conducted 4 in-depth interviews (IDIs) with teachers to collect information on their perceptions about students' knowledge regarding the relationship

For peer review only - http://bmjopen.bmj.com/site/about/guidelines.xhtml

between HWWS and STH infection; experience with implementing handwashing lessons; availability and accessibility of handwashing materials at school, and perceived effectiveness of the intervention in targeting motivational and emotional drivers for handwashing.

For the second round of data collection (October 2018), we completed 5 friendship pair interviews (FPIs) at each school, for a total of 10 respondents per school. FPI is a method used to collect data from children where the target respondent is encouraged to come to the interview with a friend in order to create a conducive, non-threatening environment for the children[25] . The research team, with support from respective class teachers, solicited student volunteers. In each class, one student was selected. The selected student was asked to request his/her best friend to join the interview. Both the FGDs and FPIs collected information on students' knowledge regarding the relationship between HWW and STH infection, perceptions about implementation of handwashing lessons (including their perception about how consistent was the delivery of the handwashing messages), availability and accessibility of handwashing materials at school, and connections between education materials and targeted motivational and emotional drivers of HWWS. Whereas FGDs were used to gain insights into children's shared understanding of how the intervention components had influenced handwashing and the ways in which individuals influenced by others in a group situation, we used FPIs to gain insight into individual children's practices and personal experiences. During the second round of data collection, we also completed semi-structured interviews with 4 teachers per school, these were the same teachers interviewed in round one.

Data was collected using pilot tested interview guide (supplementary file) . All interviews and FGDs were conducted in private empty rooms provided by the school administration.

For peer review only - http://bmjopen.bmj.com/site/about/guidelines.xhtml

Each FGD/FPI lasted between 1 hour 30 minutes and 2 hours while interviews with teachers lasted between 30 and 45 minutes. All the interviews were collected in Kiswahili, transcribed, and translated in English by a bilingual speaker for analysis.

Research team

The data collection team comprised three Tanzanian social scientists at PhD or MSc level (EO; KM, OM) and two research assistants, one male and one female with experience in qualitative research methods. Prior to starting fieldwork the research assistants received additional one week's training in qualitative research methods. The first author (EO), participated in actual data collection at the beginning of each data collection round and coordinated the study. Before each round of data collection the study team visited the schools, explained the purpose of the study, discussed study activities with the school administration and made appointments for interviews and FGDs.

Public involvement

This qualitative study is assessing an intervention that was preceded by rigorous formative research. During the formative research there was extensive involvement of students, teachers and regional and district education officers and school WASH coordinators in developing, testing and modifying the various components of the intervention. The feedback received from these stakeholders informed the design and implementation of the intervention. Similarly, the interview and FGD guides used in the qualitative study were pilot-tested and revised to include feedback from the participants. After the end of the

For peer review only - http://bmjopen.bmj.com/site/about/guidelines.xhtml

research, the findings from the main trial and from this qualitative study will be disseminated through stakeholders meetings.

Data analysis

Audio recorded interviews and FGDs were transcribed in Kiswahili and translated into English by the lead author and combined with field notes ready for analysis. Data were analyzed using Atlas.ti version 7, a qualitative data analysis and research software

The COM-B conceptual framework served as a guide for data analysis and was used to define themes in analysis. A thematic analysis approach was used to facilitate the structuring, description and interpretation of results [26] . During analysis, the first author read all interview and FGD transcripts and formulated draft codes based on recurring accounts and descriptions that were identified in the transcripts. The first and last authors reviewed, discussed, and refined these intial codes and then mapped each code back to the COM-B framework. There were no significant disagreements about how specific codes mapped back to the COM-B Framework. However, there were discussions about codes that conflated one or more COM-B determinants. These codes were then revised into sub-codes that better mapped to an individual COM-B determinant. Each code was further classified into facilitators of and barriers to HWWS. After the coding structure was refined, the first author applied the codes to all interview and FDG transcripts and prepared summary memos on specific groups of related codes.

Data from both rounds were analyzed together using the same methods. Data from each of the two rounds of data collection were then directly compared [26].

For peer review only - http://bmjopen.bmj.com/site/about/guidelines.xhtml

For peer review only

Ethical considerations

Protocol, tools, and consent forms were reviewed by the National Health Research Ethics Review Committee of Tanzania (approval number: **NIMR/HQ/R.8a/Vol.IX/2665**) and the Ethics Committee of the London School of Hygiene and Tropical Medicine (**LSHTM Ethics Ref: 14539**). All participants provided informed assent (students) and consent (teachers); and parental consent was obtained for all participating students. Permission to interview students was sought from parents/guardians, while permission to interview teachers and school staff was sought from the school authorities. Personal data was anonymised, using ID numbers and stored data was stripped of names, and password protected for use by named research staff only.

Results

A total of 60 students participated in 8 FGDs and another group of 40 students participated in 20 FPIs. On average a students FGD/FPI lasted for 1 hour 40 minutes while teachers interview was about 40 minutes. In addition, in-depth interviews were held with 16 teachers whom were interviewed two months after the start of intervention and again 8 months after intervention had been rolled out. (Table 2). All teachers and students selected for participation provided consent / assent and took part in the study.

For peer review only - http://bmjopen.bmj.com/site/about/guidelines.xhtml

Data collection round	Study participants					
	Students			Teachers		
	Male	Female	Age range	Male	Female	Age range
Round 1	30	30	7-15 years	7	9	23-52 years
Round 2	20	20	8-14 years	7	9	23-52 years

Table 2: Gender and age range of study participants

Factors that study participants identified as facilitators of good hand-washing behaviour were classified as according to the COM-B conceptual framework. Factors related to *capability* comprised knowledge and understanding of the relationship between handwashing and STH, knowledge of correct handwashing; practical skills regarding handwashing following practical demonstrations of handwashing. Factors related to *social and physical opportunity* included the successful development of a school community of students and teachers who supported and encouraged handwashing; and the improved quality, number and location of handwashing stations with access to both water and soap. Reflective and automatic factors related to increased *motivation* for handwashing comprised an increased understanding of the link between handwashing and good health that encouragds positive feeling about hand washing, disgust caused by realizing that STH infection is linked to ingesting faecal matter; the use of relatable child characters that triggered a feeling of nurture. The main barrier identified was the inconsistent availability of water and soap.

For peer review only - http://bmjopen.bmj.com/site/about/guidelines.xhtml

Capability

Two significant themes emerged from the analysis releated to handwashing capability: perceived improvements in handwashing skills and techniques and reported improvements in knowledge about when and how to wash hands.

Handwashing skills: During handwashing demonstrations, all volunteers were able to follow correct handwashing steps. Participants reported to have mastered handwashing because they had participated in practical handwashing sessions:

> *The teacher took us to the handwashing stations and showed us how to wash hands. We were told that when you come from the toilet you wet your hands, apply soap, then scrub* **(FGD older boys, school 2).**

Both teachers and students believed that the acquired skills were important in facilitating handwashing.

Handwashing lessons and educational messaging: Both children and teachers indicated that teaching about handwashing and the use of educational messaging had greatly improved children's knowledge about how and when to wash hands. All children in both the FGDs and FPIs recalled receiving at least two lessons specific to handwashing in addition to the general sanitation and hygiene education provided within their normal school curriculum. During the interviews and focus group discussions we asked students the following question: *What happens if you don't wash your hands?* Students could explain the link between hand hygiene and infections. The quote below is an example common stories throughout the students' interviews:

For peer review only - http://bmjopen.bmj.com/site/about/guidelines.xhtml

If I do not wash my hands, I get diseases especially worms. For example if I eat even a fruit without washing my hands that means I am eating dirt and I will get worms and stomach ache. So whenever you eat without washing hands you get worms **(FPI younger girls school, 3)**

Students also discussed their own behaviour related to the key times to wash hands with soap (after using the toilet and before eating), as illustrated in the following quote:

Before the project came … We did not always wash hands after using the toilet. But now were are motivated because we have been educated on the importance of handwashing so we now wash our hands always **(FPI younger girls school, 1)**

Knowledge about handwashing and key times to wash hands with soap were consistent across all interviews and FGDs. We noted no meaningful differences between students in rural and urban areas or differences between older and younger children regarding handwashing knowledge.

Opportunity

The Mikono Safi intervention influenced opportunity for handwashing in two impotant ways - changing the physical opportunity for handwashing and altering the social environment related to handwashing at schools.

Physical Opportunity: Teachers and students alike indicated that availability and location of the handwashing facilities were important facilitators of handwashing. Teachers in particular noted that every child had to pass handwashing stations on their way to and from the latrine. Handwashing stations were visible and difficult to ignore:

For peer review only - http://bmjopen.bmj.com/site/about/guidelines.xhtml

Given the location of the handwashing stations… on the way out of the toilet, everybody who uses the toilet can see them on their way out…but you know children like to play with water, so making water and soap available for children makes handwashing part of a game (IDI, **Teacher 2, school 3** *)*

*…We wash our hands at the handwashing stations near the toilet. Before they installed the handwashing tanks we never used to wash hands because we did not have water to wash hands but these days we wash our hands because they have put the tanks. But in the past we never used to wash hands (***FPI, younger girls, school 3***)*

Children in the FGDs indicated that the improved handwashing facilities were not only easier and more convenient to use when compared to the tippy-taps that the school was using before but also the tanks contained much more water allowing a larger number of students to have access to water for washing hands.

*"…Before the installation of the handwashing tanks we were using tippy-taps to wash hands and we had only few of them, the water would get finished quickly. But they were even difficult to use. With the current handwashing tanks you go there and open the tap and wash hands. The tanks are big and many students are able to wash hands using water in the tanks. (***FGD, older boys, school 1***)*

Beyond the physical availability of handwashing facilities, the most important facilitator to handwashing was the consistent provision of supplies (water and soap). In some schools, especially schools with a large student population, it was not uncommon for water to get used up early in the day - particularly during break time when the majority of the children used the toilet. Schools were able to mitigate these interruptions in water availability in

For peer review only - http://bmjopen.bmj.com/site/about/guidelines.xhtml

various ways including creating rosters for fetching water. Schools that had well supervised and well implemented class rosters for fetching water more children indicated that water was consistently available at the handwashing station.

> *…there are times when water gets finished, but in our school we have a teacher and students responsible for health and hygiene. So when water is finished from the tanks we inform the teacher or student leader and they send a group of students to bring water (***FPI, older girls, school 2)**

Water availability, however, was often determined by factors outside of the school's direct control. All schools experienced disruptions in water supply to varying degrees. In three of the four schools in the study, this was a problem that occurred during the morning break when the majority of the students used the toilets and during the dry season in which the majority of schools in the region experience water shortages. In one school this problem occurred multiple times during a week. During periods of limited availability, teachers needed to interrupt classes for certain groups of students in order for them to fill the tanks. Often, teachers were unwilling to interrupt classes, especially in schools that did not have a well-established class roster for refilling water tanks. Lack of water had a negative influence on handwashing behaviours in general, particularly if water was unavailable for more than one day in a week.

In general, both students and teachers reported that soap was not consistently provided for washing hands. This was primarily an issue of distribution rather than of availability. At one school, for example, there was no soap available for students at the handwashing station when FGD participants were asked to demonstrate behaviours. Students noted that they had reported this to the teacher responsible earlier but in vain. However, when the

For peer review only - http://bmjopen.bmj.com/site/about/guidelines.xhtml

moderator sent a student to the head teacher's office, the soap was provided. Similarly, discussions with school administrators indicated that, when provision of soap was inconsistent, it was usually available at the store. :

> *"There are a number of times during the week when soap is not available at the handwashing station. This can happen like three times in a week. If the soap is not at the handwashing station we usually go to the Head teacher's office to get the soap"* (**FGD, younger girls, school 3**)

> *There are times when the soap is not available because the teacher responsible did not check or the students have not reported. This happens sometimes but that usually is for a few hours in a day, not the whole day* (IDI, **Teacher 1, school 2)**

Social opportunity: Data suggest that the Mikono Safi intervention had created school communities that valued handwashing and that teachers were willing to support handwashing activities through teaching and reminding children to wash hands, supervising children to fill the water tanks, and ensuring that soap was available. Students were also willing to participate in activities that ensured water and soap were available at the handwashing stations:

> *"If I came out of the toilet and there is no water I will pick a bucket get water from the main reserve tank and fill the handwashing tanks so that other children and I can wash our hands" (***FGD, Older girls, school 2***)*

For peer review only - http://bmjopen.bmj.com/site/about/guidelines.xhtml

"Today I went to the toilet in the morning but there was no soap at the handwashing station at that time. I reported to the student in charge of sanitation and s/he promised to inform the teacher that there was not soap at the handwashing station" (**FPI, older girls, school 1**)

Students, especially the youngest ones, were keen to report colleagues that did not wash hands after using the toilet. Teachers described experiences where students would report to them other students who did not wash hands.

"Certainly, there are changes in children's handwashing behaviour. I am a teacher for the pre-primary class (the youngest group in school). What I have observed is that these days these young ones report colleagues that do not wash hands after using the toilet. If one of them used a toilet and did not wash hands the others will come running to you to report him or her; they will come and tell you 'teacher, so and so did not wash his/her hands after using the toilet'; they give you such reports (**IDI, teacher, school 2**)

Motivation

Automatic motivation/Emotional triggers: Data suggest that the intervention was successful in reaching targeted emotional drivers of disgust, fear, and nuture in children. In particular, many children described Muta – the boy character depicted in the educational materials as a boy who did not know how to wash his hands - as repulsive and disgusting. On the other hand children found Koku (portrayed as a hygiene loving child) attractive and

For peer review only - http://bmjopen.bmj.com/site/about/guidelines.xhtml

every child interviewed indicated that he or she would like be like Koku. Koku was described as clean and smart, but also kind and willing to teach a fellow student who may not have knowledge of handwashing yet.

> *I feel bad about Muta's behaviour; the fact that he did not wash his hand makes me feel disgusted…. but we like Koku because she was clean and she knew how to wash hands in order to protect herself from disease. Everyone would like to be like Koku, she is clean, smart and she likes helping others* (**FGD, older girls, school 4**)

> *… the teachers told us that if you use the toilet but you do not wash your hands and you go and eat anything you may be eating feces. So when you think about that you remember to wash your hands and you will remind all your friends to wash hands (**FGD, older girls, school 1**)*

Reflective motivation: Although introducing class rosters for fetching water was not part of the original intervention package, two of the four schools used rosters to ensure water was available at the handwashing facilities. For some schools this activity was led by students lead and for others by a teacher. For example, in schools that had active students' health clubs, members of these clubs were charged with the responsibility for supervising all health related activities in school. Hence, schools which had active student health clubs developed the roster for health activities for each day of the week.

Students in schools that had student leaders involved in taking decisions about replenishing water and soap were more motivated to take action to ensure they have materials to wash hand. When children believed that they had power to influence their own handwashing

For peer review only - http://bmjopen.bmj.com/site/about/guidelines.xhtml

practices –- they were willing to take an active role in activities that would facilitate handwashing among themselves and others.

> *"The students have received the handwashing lessons very well. They wash hand but they also encourage others to wash hands. For example if they saw a colleague who is dirty they encourage him/her to wash hand in order to avoid being labeled Muta, the character depicted in some of the education materials of a dirty boy…"* (**IDI, teacher 1, school 3**)

Changes over time: Results from the two rounds of data collection were compared to detect possible differences in terms of perceived barriers and facilitators related to capability, opportunity and motivation for handwashing. We did not find any differences. In both round one and two, students demonstrated good knowledge and skills for handwashing. Similarly, the opportunity for handwashing remained largely unchanged between the data collection points at round one and two. The installed handwashing facilities remained well maintained in all four schools. The schools continued to experience disruptions in water supply, and as before success in mitigating such disruptions varied from school to school.

Table 3 summarises capability, opportunity, and motivational determinants of HWWS based associated with the Mokono Safi Intervention based on student and teacher reported data.

For peer review only - http://bmjopen.bmj.com/site/about/guidelines.xhtml

Determinant		Facilitators	Barriers
Capability	Psychological	Knowledge about the relationship between HWWS and STH<br><br>Knowledge of key moments for hand hygiene	
	Physical	Skills developed through HWWS demonstration and rehearsal	
Opportunity	Social	Teachers support and encourage HWWS<br><br>Peers who support HWWS	
	Physical	Improved quality, quantity, and location of HW stations	Inconsistent availability of water<br><br>Inconsistent availability of soap at HW stations
Motivation	Automatic	Emotional response (disgust / fear) triggered through messages and content<br><br>Relatable characters inspiring feelings of nurture among older students<br><br>Reminders (nudges) that reinforced handwashing habits	
	Reflective	Proactive student engagement in water collection and soap provision	

Table 3: Capability, Opportunity, and Motivational Determinants of HWWS as reported by the participants

For peer review only

For peer review only - http://bmjopen.bmj.com/site/about/guidelines.xhtml

Discussion

Our study set out to describe the barriers and facilitators to handwashing behaviour from the perspectives of teachers and students participating in the Mikono Safi intervention trial. Our results show that participants associated the intervention with improved skills and knowledge. Children's description of the intervention matched with the targeted emotional drivers of behaviours. Both students and teachers described changes in the social environment of the school that fostered better HWWS behaviours. However, the physical opportunity for handwashing was mixed. When both water and soap were available, the new handwashing stations increased the ease and convenience of handwashing among students. On the other hand, we noted that schools reported on-going challenges with water availability and that the provision of soap at handwashing stations – even when available at the school – remained inconsistent.

Our data suggest that children receiving the Mikono Safi intervention had high capability for HWWS. Students could both describe and demonstrate proper hand hygiene and reported knowing when and why hands should be washed with soap. Although insufficient on its own, knowledge may be a necessary precursor for HWWS [27]. It is important to note that education and health messaging included in the Mikono Safi intervention were not abstract – they were focused around the risk of preventing intestinal worms. Studies in India have shown that providing individuals with specific- and actionable health information can improve WASH behaviours [27]. Having lessons that focus on hand hygiene and its risk with specific health outcomes in addition to regular sanitation and hygiene education may be a promising option for facilitating handwashing behaviour among school children.

For peer review only - http://bmjopen.bmj.com/site/about/guidelines.xhtml

The qualitative data from students and teachers suggest that the Mikono Safi intervention was associated with the targeted emotional drivers of nurture, fear, and disgust. In the student data, there were rich stories of children describing Muta's poor hygiene as repulsive and disgusting. Students noted that they often washed hands to avoid being labelled "Muta" by their colleagues. Studies have shown how motivational drivers can be successful in changing hygiene behaviours [28], although only a limited number of studies have explored handwashing motives related to children. The SuperAmma study in India [28] demonstrated significant improvement in hygiene behaviours based on messaging that targeted motivational drivers in isolation from health education. Our study shows how these emotional drivers can be combined with targeted health education to influence both motivation and capability.

Our findings suggest that the nurture motive was particularly salient to students. Students spoke fondly of the fact that Koku (the female student appearing in education materials) helped her friends out; and both teachers and students discussed the ways that the school had provided a supportive environment for handwashing. The salience of the nuture motive in our study population is consistent with other studies of student handwashing. For example, a study in Bangladesh that used covert video cameras to record handwashing behaviour found numerous instances of older children helping younger children washing hands and modelling positive behaviours for one another [29]. Students in our study responded positively to the nurture messages, suggesting that positive peer pressures are a potential avenue for improving HWWS in schools.

For peer review only - http://bmjopen.bmj.com/site/about/guidelines.xhtml

Making handwashing facilities visible, difficult to ignore but also convenient, were among the attributes mentioned as important by both teachers and students. However, beyond the physical positioning and convenience, the most important facilitator to handwashing was consistent provision of water and soap. Although all schools experienced disruptions in water supply especially at peak hours, some schools that were able to implement a well supervised class rosters for fetching water succeeded to avoid interruptions in water availability at handwashing stations. Similarly students and teachers suggested that inconsistency in the availability of soap at the handwashing stations was largely an issue of internal distribution rather than of external logistics. Our data suggests that while the Mikono Safi intervention was successful in changing capability and motivation, the opportunity for HWWS may not have been sufficiently improved in some schools, either due to external factors such as the dry season, or to internal factors such as the lack of class duty rosters for the collection of water or soap.

Our study has some limitations : first, this was a qualitative study involving a small sample of schools, teachers and pupils, and therefore our results may not be generalizable to other intervention schools. However, our schools were purposively selected to reflect the general diversity of schools included in the Mikono Safi study and are similar to most schools in the Kagera region. Secondly, findings are primarily based on individual interviews and FGDs. Data from direct observation of students' behaviour was not collected during the qualitative study. However, FGDs, FPIs with students and IDIs with teachers proved to be well suited methods to examine barriers and facilitators to appropriate handwashing behaviour, and produced consistent results. We also do not have information on drivers of behaviour before the intervention was implemented and were not able to interview students in

For peer review only - http://bmjopen.bmj.com/site/about/guidelines.xhtml

enrolled control groups and therefore can only draw associations between the intervention and students reported determinants of handwashing. Students and teachers were aware of our affiliation with the larger Mikono Safi intervention trial and courtesy bias may have influenced both teachers and students to provide answers they assumed we wanted to hear. Reports on changes in behaviour should therefore be interpreted with caution. However, the majority of our study explored the drivers of behaviour rather than the behaviours themselves. We also found high consistency in responses from both students and teachers, and respondents were forthcoming about challenges related to sustained soap provision at schools.

For peer review only

The COM-B Framework provided a prudent way to explore the success of the Mikono Safi intervention in addressing the barriers and facilitators for handwashing in our study population. Our analysis was based on drivers and facilitators among students. Viewed from an instititutional perspective, several of our key findings could relate to other COM-B determinants. For example, consistent provision of soap from the institution's perspective may be a factor of reflective motivation – schools may not have prioritized soap distribution or they could have made conscious decisions to without materials from students out of budget or supply concerns. Given the important role in intervention compliance in several school-based WASH interventions[11, 14], more theoretically informed research is needed to unpack the drivers of institutional factors that contribute to behavioural outcomes.

In conclusion, the Mikono Safi intervention was associated with high motivation and capability for handwashing, but ensuring the opportunity for handwashing remained a challenge at some schools, especially with respect to a consistent availability of water. For such schools a much stronger multi-sectoral collaboration between the departments for

For peer review only - http://bmjopen.bmj.com/site/about/guidelines.xhtml

education and water may be required. Results from the forthcoming trial will allow us to understand the impact this intervention had on child health outcomes. Future research focused on hygiene and hygiene behaviour change should further explore the ways in which motivational and educational messaging can change specific determinants of behaviour; new intervention modalities that promote school-level adherence to routine provision of basic supplies need development and testing in resource-scarce environments like Tanzania

For peer review only

For peer review only - http://bmjopen.bmj.com/site/about/guidelines.xhtml

References

1. WHO *Soil-transmitted helminth infections updates, in facts sheet*. 2019. https://www.who.int/news-room/fact-sheets/detail/soil-transmitted-helminth-infections.
2. Drake, L.J., et al., *Geohelminth infections (ascariasis, trichuriasis, and hookworm): Cognitive and developmental impacts.* Seminars in Pediatric Infectious Diseases, 2000. **11**(4): p. 245-251.
3. Hotez, P.J., et al., *Hookworm Infection.* New England Journal of Medicine, 2004. **351**(8): p. 799-807.
4. WHO, *Preventive chemotherapy in human helminthiasis. Coordinated use of antihelminthic drugs in control interventions: a manual for health professionals and programme managers*. 2006, World Health Organization: Geneva.
5. MOHSW, *Tanzania National Master Plan for Neglected Tropical Diseases*. 2009, Tanzania Ministry of Health and Social Welfare.
6. GAHI, *Distribution of soil-transmitted helminth survey data in Tanzania*. 2017, Global Atlas of helminth infections.
7. WHO *Soil-transmitted helminth infections, in Fact sheet* 2013b. https://www.who.int/news-room/fact-sheets/detail/soil-transmitted-helminth-infections.
8. Yap, P., et al., *Rapid Re-Infection with Soil-Transmitted Helminths after Triple-Dose Albendazole Treatment of School-Aged Children in Yunnan, People's Republic of China.* The American journal of tropical medicine and hygiene, 2013. **89**.p.23-31
9. Caruso, B.A., et al., *Assessing the impact of a school-based latrine cleaning and handwashing program on pupil absence in Nyanza Province, Kenya: a cluster-randomized trial.* Tropical Medicine & International Health, 2014. **19**(10): p. 1185-1197.
10. Chard, A.N., et al., *The impact of school water, sanitation, and hygiene improvements on infectious disease using serum antibody detection.* PLOS Neglected Tropical Diseases, 2018. **12**(4): p. e0006418.
11. Dreibelbis, R., et al., *The Impact of School Water, Sanitation, and Hygiene Interventions on the Health of Younger Siblings of Pupils: a Cluster-Randomized Trial in Kenya.* American Journal of Public Health, 2013. **104**(1): p. e91-e97.
12. Freeman, M., et al., *The impact of improved school water, sanitation and hygiene access on pupil diarrhea: A cluster randomized trial.* Am J Trop Med Hyg, 2012. **87**(Supplement 1): p. 428.
13. Freeman, M.C., et al., *Systematic review: Hygiene and health: systematic review of handwashing practices worldwide and update of health effects.* Tropical Medicine & International Health, 2014. **19**(8): p. 906-916.
14. Chard, A.N. and M.C. Freeman, *Design, Intervention Fidelity, and Behavioral Outcomes of a School-Based Water, Sanitation, and Hygiene Cluster-Randomized Trial in Laos.* International Journal of Environmental Research and Public Health, 2018. **15**(4): p. 570.
15. Garn, J., et al., *The Role of Adherence on the Impact of a School-Based Water, Sanitation, and Hygiene Intervention in Mali.* The American Journal of Tropical Medicine and Hygiene, 2017. **96**: p. 16-0558.
16. Dreibelbis, R., et al., *Behavior Change without Behavior Change Communication: Nudging Handwashing among Primary School Students in Bangladesh.* International journal of environmental research and public health, 2016. **13**(1): p. 129.
17. Aunger, R. and V. Curtis, *Behaviour Centred Design: towards an applied science of behaviour change.* Health psychology review, 2016. **10**: p. 1-22.
18. Kapp, K., *The gamification of learning and instruction: game-based methods and strategies for training and education*. 2012: John Wiley & Sons.

For peer review only - http://bmjopen.bmj.com/site/about/guidelines.xhtml

19. Watson, J., et al., *Child's play: Harnessing play and curiosity motives to improve child handwashing in a humanitarian setting.* International Journal of Hygiene and Environmental Health, 2019. **222**(2): p. 177-182.
20. Grover, E., et al., *Comparing the behavioural impact of a nudge-based handwashing intervention to high-intensity hygiene education: a cluster-randomised trial in rural Bangladesh.* Tropical Medicine & International Health, 2018. **23**(1): p. 10-25.
21. Coombes, Y. and J. Devine, *Introducing FOAM: A Framework to Analyze Handwashing Behaviors to Design Effective Handwashing Programmes* 2010, World Bank: Washington, DC.
22. Dreibelbis, R., et al., *The Integrated Behavioural Model for Water, Sanitation, and Hygiene: a systematic review of behavioural models and a framework for designing and evaluating behaviour change interventions in infrastructure-restricted settings.* BMC Public Health, 2013. **13**(1): p. 1015.
23. Mosler, H.-J., *A systematic approach to behavior change interventions for the water and sanitation sector in developing countries: a conceptual model, a review, and a guideline.* International Journal of Environmental Health Research, 2012. **22**(5): p. 431-449.
24. Michie, S., M.M. van Stralen, and R. West, *The behaviour change wheel: A new method for characterising and designing behaviour change interventions.* Implementation Science, 2011. **6**(1): p. e1-e11
25. Wilson, A., A. Onwuegbuzie, and L.P. Manning, *Using paired depth interviews to collect qualitative data.* The Qualitative Report, 2016. **21**(9): p. 1549-1573.
26. Attride-Stirling, J., *Thematic networks: an analytic tool for qualitative research.* Qualitative Research, 2001. **1**(3): p. 385-405.
27. Trent, M., et al., *Access to Household Water Quality Information Leads to Safer Water: A Cluster Randomized Controlled Trial in india.* Environmental Science & Technology, 2018. **52**(9): p. 5319-5329.
28. Biran, A., et al., *Effect of a behaviour-change intervention on handwashing with soap in India (SuperAmma): a cluster-randomised trial.* The Lancet Global Health, 2014. **2**(3): p. e145-e154.
29. Grover, E., et al., *Social Influence on Handwashing with Soap: Results from a Cluster Randomized Controlled Trial in Bangladesh.* The American Journal of Tropical Medicine and Hygiene, 2018. **99**. p.934-936

For peer review only

For peer review only - http://bmjopen.bmj.com/site/about/guidelines.xhtml

Footnotes

Acknowledgments: We thank John Robert and Winnie Muriba for assisting with data collection and transcription of the interviews and focus group discussions. We are grateful to the parents for allowing their children to take part in the study and the school administration of the 4 schools for giving us permission to talk to students and teachers. We are grateful to the participating students and teachers for their time and contributions to the study. This study was funded by the Department of International Development (DFID) of the United Kingdom (UK) through the SHARE Consortium programme, based at the London School of Hygiene and Tropical Medicine (LSHTM).

Contributors: EO conceived the study and was the investigator with overall responsibility for the management of the study. EO and RD were responsible for the original design and protocol. SK, HG, SK, contributed to the study design methodology and obtaining funding. KM and OM supervised fieldwork and reviewed the draft manuscript. EO analyzed the data and wrote the first draft of the manuscript. RD, HG, SK, and SK provided a critical review for important intellectual content. All authors were involved in the final version of the manuscript and approved the manuscript for submission.

Disclaimer: The views expressed are those of the authors and not necessarily those of the funder

Competing interests statement: There are no competing interests for any authors

Data sharing statement: The data are anonymised transcripts and are available upon reasonable request by emailing elialilia@mitu.or.tz..

For peer review only

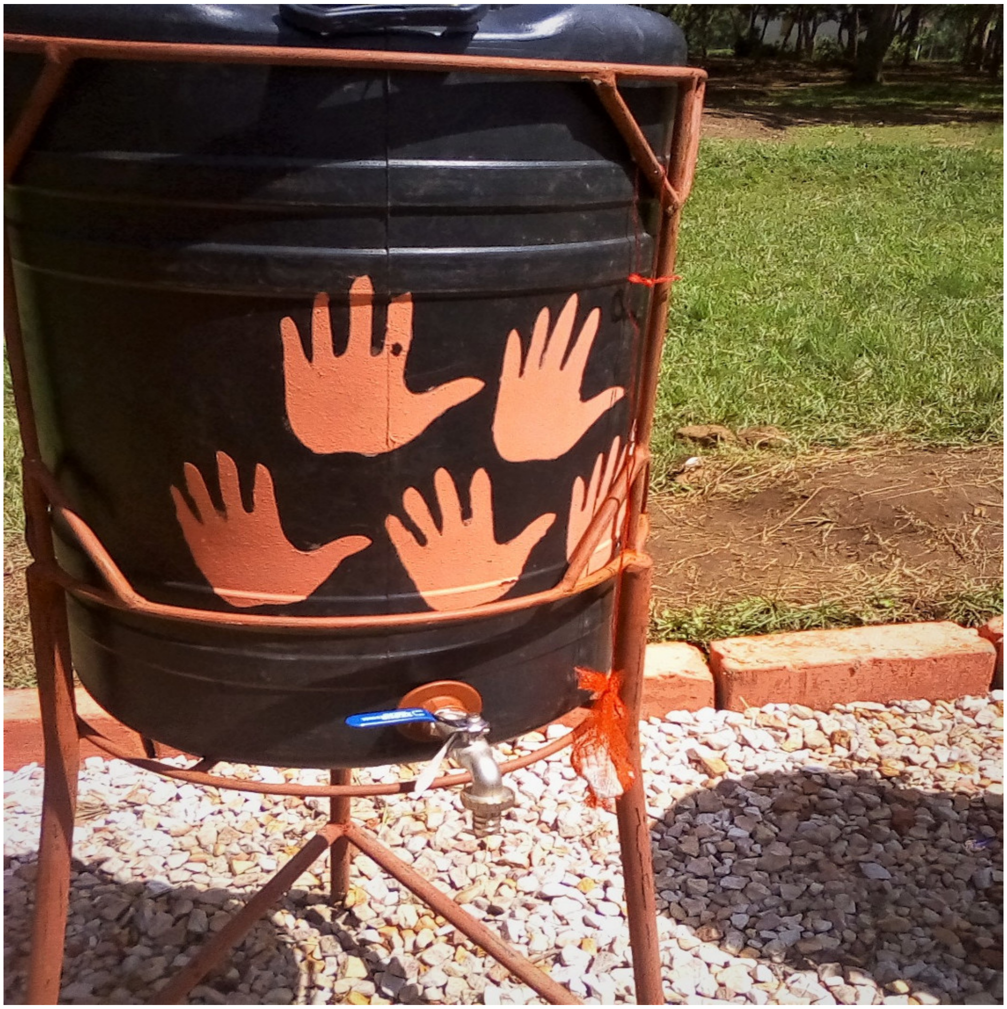

Figure 1: Handwashing facility with “nudges” installed in the intervention schools

STUDY GUIDES

Appendix 1: FGD/Friendship Pair Interview question guide for students

Name of District....................................... Name of School..

Age of the respondent............................ Class of the respondent..........................

A: Knowledge about HWWS and helminth infections

1. Tell me about the last time you washed your hands.
    a. What did you use?
    b. Where were you? (*School, home, friend's house, etc*.)
    c. What were you doing immediately before you washed your hands?
    d. What did you do after you washed your hands?
    e. Potential probes:
        i. Are there other times during the day you usually wash your hands?
        ii. Why is it important to wash hands then?
2. The last time you washed your hands; can you show me how you did it? (consider bringing soap and water to interview)
    a. Is that how you always wash your hands? [*Probe on any different times or ways that they describe].*
3. What happens if you don't wash your hands?[*Probe on responses]*
4. Imagine you are at school and you go to wash your hands.
    a. You find there is no soap at the handwashing station. What would you do? (*Probe on responses: Then what would you do? Would you try something else? Etc*.)
    b. Now imagine there is no water at the handwashing station. What would you do then? (Probe…)
5. Now, imagine you are at home and want to wash your hands. What do you do if there is no soap? No water?

B: Implementation of handwashing lessons

6. Please tell me about any lessons you've had in class recently about handwashing
7. What do you remember about the lesson? (Probe on as many specifics as possible.)
8. During the lesson,
    a. What did the teacher do?
    b. What did the children do?
9. What parts did you like most about the lesson? Why? What was your favourite part of the lessons?

For peer review only - http://bmjopen.bmj.com/site/about/guidelines.xhtml

C: Availability and accessibility of Handwashing materials

10. While in school, where do you go to wash your hands?
11. Are there times when you want to wash your hands but water is finished? If yes, tell me about the last time you went but the water was finished. (Probe on how long ago, what time of day, what they did in response.) How often does this happen?
12. Are there times when you need soap to wash your hands but soap is finished? Same as above.

D: Effectiveness of Mikono Safi in targeting in motivational and emotional drivers of handwashing with soap

13. During the handwashing exercise that we talked about earlier, teachers used some examples,
    a. What are the examples?
    b. Which of the examples do you remember most?
    c. Why do you remember that example?
14. [Bring and use some of the motivational messages during the interviews; Show the children the Muta and Koku flip chart then ask]
    a. Please tell me what you see on the flip chart
    b. What do you think about Muta?
    c. How does Muta's poor hygiene behavior make you feel?
    d. What do think about Koku?
    e. How does Koku's good hygiene make you feel?
    f. Imagine Muta was you young brother, what would to help him?
15. Show the children show the children the side by the side poster
    a) What do you see on the poster? [Probe on the response]
    b) Focusing on the side of the child with worms
        i. What is the girl/boy doing wrong?
        ii. How does the girl/boy's behavior make you feel?
        iii. What would you do if you were this girl/boy?
        iv. What would you help if this boy/girl was your little brother/sister?
16. If you saw child from standard one coming from the toilet and running to play, what would you do? Why?
17.

E: Consistence in message delivery

18. Which day of the week do you usually receive the hand washing lessons?
19. What time of the day do the teachers come for hand washing lessons?
20. What activities do children participate in during handwashing lessons?

For peer review only - http://bmjopen.bmj.com/site/about/guidelines.xhtml

Appendix 2: In-depth Interview guide for Teachers/school staff

Name of District....................................... Name of School..
Age of the respondent............................ Education level of the respondent.........................

A: Knowledge about HWWS and helminth infections

1. In your opinion, how much do children know about when and how to washing hands? [*probe on response*]
2. What do you think motivates them to wash hands?
3. What do you think discourages from washing hands at critical times?
4. In your observation, If for example, children want to wash hands but there is no water, what do they usually do?
5. In your observation, If for example, children want to wash hands but there is no water, what do they usually do?

B: Lessons implemented and appropriateness of the lessons

6. Please tell me how handwashing lessons are delivered in your school
   a. What has been your role in delivering these lessons?
   b. In what classes are the lessons delivered?
   c. How often are these lessons delivered?
   d. What activities are the children involved in during these lessons?
   e. What has been your experience in delivering these lessons?
7. How would you describe the involvement of teachers and school staff in delivering handwashing lessons in your school? [*probe on the answer*]
8. What motivates teachers to get involved in delivering handwashing lessons?
9. What discourages teachers from getting involved in delivering handwashing lessons
10. What is your opinion about the contents of the handwashing curriculum?
11. How far if the curriculum is able address knowledge and skill gaps related to handwashing in children?
12. Do you have any recommendations to improve the handwashing curriculum?

C: Availability and accessibility of Handwashing materials

13. Tell me about the availability of water and soap in your school
    a. How does the school ensure water is available for children to wash hands?
    b. How does the school ensure soap is available for children to wash hands?
14. Are there times when water is not available for children to wash hands? How often does this happen?
15. Are there times when soap is finished? How often does this happen?
16. How does the school maintain the handwashing stand?

For peer review only - http://bmjopen.bmj.com/site/about/guidelines.xhtml

17. How does your school ensure that latrines are kept clean? [*probe for who is involved*]
18. How does your school ensure there are sufficient and appropriate supplies for latrine cleaning?
19. How are latrines managed and maintained?[probe about who is involved]

D: Effectiveness of Mikono Safi in targeting in motivational and emotional drivers of handwashing with soap

20. How would you describe children response to handwashing lessons?
21. Among the motivational messages used in the handwashing curriculum, what parts do children enjoy most during handwashing lessons? Why?
22. In your view, motivational messages; what parts do children remember most about handwashing lessons? Why?
23. What changes have you observed in children's handwashing behavior?
    a. Is there a difference between girls and boys?
    b. Are there differences among age groups?

For peer review only - http://bmjopen.bmj.com/site/about/guidelines.xhtml

COREQ (COnsolidated criteria for REporting Qualitative research) Checklist

A checklist of items that should be included in reports of qualitative research. You must report the page number in your manuscript where you consider each of the items listed in this checklist. If you have not included this information, either revise your manuscript accordingly before submitting or note N/A.

Topic	Item No.	Guide Questions/Description	Reported on Page No.
**Domain 1: Research team and reflexivity**			
Personal characteristics			
Interviewer/facilitator	1	Which author/s conducted the interview or focus group?	9
Credentials	2	What were the researcher's credentials? E.g. PhD, MD	9
Occupation	3	What was their occupation at the time of the study?	9
Gender	4	Was the researcher male or female?	9
Experience and training	5	What experience or training did the researcher have?	9
Relationship with participants			
Relationship established	6	Was a relationship established prior to study commencement?	9
Participant knowledge of the interviewer	7	What did the participants know about the researcher? e.g. personal goals, reasons for doing the research	9
Interviewer characteristics	8	What characteristics were reported about the inter viewer/facilitator? e.g. Bias, assumptions, reasons and interests in the research topic	9
**Domain 2: Study design**			
Theoretical framework			
Methodological orientation and Theory	9	What methodological orientation was stated to underpin the study? e.g. grounded theory, discourse analysis, ethnography, phenomenology, content analysis	4, 9, 10
Participant selection			
Sampling	10	How were participants selected? e.g. purposive, convenience, consecutive, snowball	7
Method of approach	11	How were participants approached? e.g. face-to-face, telephone, mail, email	9
Sample size	12	How many participants were in the study?	7 & 10
Non-participation	13	How many people refused to participate or dropped out? Reasons?	9
Setting			
Setting of data collection	14	Where was the data collected? e.g. home, clinic, workplace	9
Presence of non-participants	15	Was anyone else present besides the participants and researchers?	9
Description of sample	16	What are the important characteristics of the sample? e.g. demographic data, date	10
Data collection			
Interview guide	17	Were questions, prompts, guides provided by the authors? Was it pilot tested?	9
Repeat interviews	18	Were repeat inter views carried out? If yes, how many?	8
Audio/visual recording	19	Did the research use audio or visual recording to collect the data?	9
Field notes	20	Were field notes made during and/or after the inter view or focus group?	9
Duration	21	What was the duration of the inter views or focus group?	9
Data saturation	22	Was data saturation discussed?	__
Transcripts returned	23	Were transcripts returned to participants for comment and/or	__

For peer review only

For peer review only - http://bmjopen.bmj.com/site/about/guidelines.xhtml

Topic	Item No.	Guide Questions/Description	Reported on Page No.
		correction?	
**Domain 3: analysis and findings**			
Data analysis			
Number of data coders	24	How many data coders coded the data?	10
Description of the coding tree	25	Did authors provide a description of the coding tree?	—
Derivation of themes	26	Were themes identified in advance or derived from the data?	10
Software	27	What software, if applicable, was used to manage the data?	9
Participant checking	28	Did participants provide feedback on the findings?	—
Reporting			
Quotations presented	29	Were participant quotations presented to illustrate the themes/findings? Was each quotation identified? e.g. participant number	11-18
Data and findings consistent	30	Was there consistency between the data presented and the findings?	11-18
Clarity of major themes	31	Were major themes clearly presented in the findings?	11-18
Clarity of minor themes	32	Is there a description of diverse cases or discussion of minor themes?	11-18

Developed from: Tong A, Sainsbury P, Craig J. Consolidated criteria for reporting qualitative research (COREQ): a 32-item checklist for interviews and focus groups. *International Journal for Quality in Health Care*. 2007. Volume 19, Number 6: pp. 349 – 357

Once you have completed this checklist, please save a copy and upload it as part of your submission. DO NOT include this checklist as part of the main manuscript document. It must be uploaded as a separate file.

For peer review only
